# Implementation of policy and management interventions to improve health and care workforce capacity to address the COVID-19 pandemic response: a systematic review

**DOI:** 10.1186/s12960-023-00856-y

**Published:** 2023-10-10

**Authors:** Ana Paula Cavalcante de Oliveira, Mariana Lopes Galante, Leila Senna Maia, Isabel Craveiro, Alessandra Pereira da Silva, Ines Fronteira, Raphael Chança, Giorgio Cometto, Paulo Ferrinho, Mario Dal Poz

**Affiliations:** 1 Instituto de Medicina Social, Universidade do Estado do Rio de Janeiro, Rua São Francisco Xavier 524-7º andar, Blocos D e E-Maracanã, Rio de Janeiro, RJ 20550-013 Brazil; 2 Global Health and Tropical Medicine, Instituto de Higiene e Medicina Tropical, NOVA University of Lisbon, Rua da Junqueira, 100, 1349-008 Lisbon, Portugal; 3 National School of Public Health, Public Health Research Centre, Comprehensive Health Research Center, NOVA University of Lisbon, Avenida Padre Cruz, 1600-560 Lisbon, Portugal; 4 Instituto Nacional de Cancer, Ministério da Saúde, Rua Marquês de Pombal, 125, Centro, Rio de Janeiro, RJ 20230240 Brazil; 5 Health Workforce Department, World Health Organization, Av. Appia 20, 1202 Geneva, Switzerland

**Keywords:** COVID-19, Health and care workforce policy, Health and care workforce capacity, Health and care workforce interventions, SARS-CoV-2

## Abstract

**Background:**

The COVID-19 pandemic highlighted pre-existing weaknesses in health and care systems and services and shortages of health and care workers (HCWs). As a result, policymakers needed to adopt measures to improve the health and care workforce (HCWF) capacity. This review aims to identify countries’ range of policies and management interventions implemented to improve HCWs’ capacity to address the COVID-19 pandemic response, synthesize their evidence on effectiveness, and identify gaps in the evidence.

**Methods:**

The literature was searched in PubMed, Embase, Scopus, LILACS–BVS, WHO’s COVID-19 Research Database and the ILO, OECD and HSRM websites for literature and documents published between January 2020 and March 2022. Eligibility criteria were HCWs as participants and policy and management interventions aiming to improve HCWF capacity to address the COVID-19 pandemic response. Risk of bias was assessed with Joanna Briggs Institute (JBI) Critical Appraisal Tools (CAT) and certainty of the evidence in presented outcomes with GRADE.

**Results:**

The searches retrieved 3378 documents. A total of 69 were included, but only 8 presented outcomes of interventions implemented. Most of the selected documents described at least one intervention implemented by countries at the organizational environment level to increase the flexibility and capacity of the HCWF to respond to the pandemic, followed by interventions to attract and retain HCWs in safe and decent working environments. There was a lack of studies addressing social protection, human resources for health information systems, and regarding the role of community health workers and other community-based providers. Regarding the risk of bias, most of documents were rated as medium or high quality (JBI’s CAT), while the evidence presented for the outcomes of interventions was classified as mostly low-certainty evidence (GRADE).

**Conclusions:**

Countries have implemented various interventions, some innovative, in response to the pandemic, and others had their processes started earlier and accelerated by the pandemic. The evidence regarding the impact and efficacy of the strategies used by countries during the pandemic still requires further research.

**Supplementary Information:**

The online version contains supplementary material available at 10.1186/s12960-023-00856-y.

## Background

The COVID-19 pandemic has challenged health and care systems (HCS) worldwide, claiming almost 7 million lives and affecting about 670 million people until 22nd May 2023 [1]. With almost all countries stricken by the pandemic facing a surge in patient cases and disruptions of essential health services, health and care workforce (HCWF) issues were the most significant barrier to scaling up access to interventions against COVID-19 [2].

According to WHO COVID-19 detailed surveillance dashboard [3], 8.977 health and care workers (HCWs) lost their lives and 5.439.192 were infected with COVID-19 between 30th December 2019 and 9th January 2023 (considering 150 countries for data regarding infection and 65 countries for deaths), though these figures reflect incomplete reporting and higher estimates of health worker deaths have been developed [4]. In addition, HCWs faced fatigue due to an increased workload (accentuated by absenteeism and quarantine) and were exposed to work-related health hazards and their consequences, including psychosocial stress, despair, violence and shortage of personal protective equipment (PPE) [5, 6], resulting in an increased number of HCWs going on strike or leaving the workforce [7, 8]. Furthermore, female HCWs, who provide the majority of care in all settings and already experience barriers at work that are not faced by their male colleagues [9], encountered additional disproportionate risks to their health and well-being during the COVID-19 pandemic [10].

The pandemic of COVID-19 highlighted foundational gaps and pre-existing weaknesses in HCS: the lack of an effective health emergency management system, including education, basic training and professional development in emergency preparedness and response for HCWs and managers [11]; and a shortage of HCWs as well as other imbalances (e.g., geographical, by service, and skill mix), longstanding challenges observed in almost every country [12, 13]. In addition, policymakers had to establish new tactics to improve the surge capacity of the HCWF to respond adequately to the needs that the pandemic brought to the fore.

The World Health Organization (WHO) synthesized evidence and contributed guidance to inform the decision-making process to "assist health managers and policy-makers at national, subnational, and facility levels in designing, managing and preserving the workforce necessary to manage the COVID-19 pandemic and maintain essential health services" [14]. However, as the epidemic spread, innovations were put in place, and updated evidence was published, access to this information became essential to inform the development and implementation of policy and interventions.

In this paper, we report on a literature review addressing two questions (Table 1) aimed at identifying countries’ range of policies and management interventions implemented to improve HCWs’ capacity to respond to the pandemic, synthesize their evidence on effectiveness, and identify knowledge gaps.

**Table 1:** Review questions for policy and management interventions to improve health and care workforce capacity to address the COVID-19 pandemic response

Review questions
1. What are the policies and management interventions implemented by countries to improve health and care workforce capacity to address the COVID-19 pandemic response?
2. What is the effectiveness of these policies and management interventions on the availability and accessibility of health and care workers to address the COVID-19 pandemic response?

## Methods

The review was structured using PICOC (Population; Intervention; Comparison; Outcomes; Context) search tool [15] (Additional File 1) and the protocol is available at Prospero (registration number CRD42022327041 at https://www.crd.york.ac.uk/prospero/display_record.php?ID=CRD42022327041).

### Eligibility criteria

The review included documents in which HCWs (population), defined as all those employed in “human health and social activities” as classified by the International Standard Industrial Classification of All Economic Activities [6, 16], working in health facilities offering all levels of care, and various employment settings. The intervention(s) studied were policy and management interventions aiming to improve HCWF capacity to address the COVID-19 pandemic response, whether implemented at the supranational national or state level, either comparing to no-intervention or to other interventions.

The main outcomes considered, when available, were the following: HCWF availability, distribution, performance, efficiency, productivity, retention, protection, working conditions and satisfaction. Additional outcomes cover absenteeism, deaths, infection, cases of violence and harassment, turnover, intention to leave, workplace hazards, financial protection, service delivery disruptions/disrupted access to essential health services (continuity of treatment of chronic diseases) and coverage.

The literature included studies (qualitative, observational, experimental, quasi-experimental, mixed methods), reviews and grey literature (technical and political documents), published between 1st of January 2020 and 1st of March 2022 in English, French, Hindi, Italian, Portuguese, and Spanish.

### Information sources and search strategy

The following literature databases were used: PubMed, Embase, Scopus, LILACS–BVS and WHO COVID-19 Research Database [17]. International organizations’ websites were also searched, namely, International Labour Organization (ILO) [18], Economic Co-operation and Development (OECD) [19] and the Health System Response Monitor (HSRM) [20]. To identify the search terms, the controlled health vocabularies DeCs (Descriptors in Health Sciences), MeSH (Medical Subject Headings) and Emtree (Embase Subject Headings) were consulted. Relevant words that were not captured in the keyword search were included as free terms. The search strategy conducted from late March to April 2022 is detailed in Additional file 2.

### Selection of studies

**Fig. 1:**
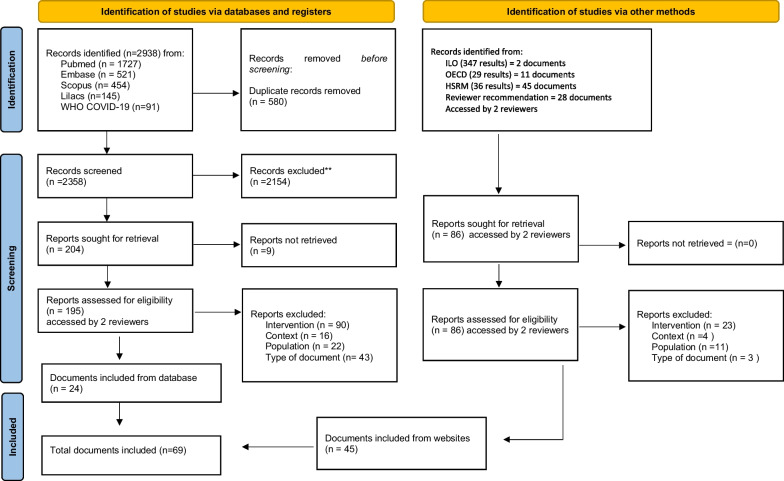
PRISMA flow diagram

EndNote [21] was used to collect, organize and manage references retrieved from the searches and to remove duplicates. Once this phase was completed, references were uploaded to Rayyan [22] to remove remaining duplicates and to apply eligibility/exclusion criteria to the title and abstracts (first phase), followed by a full-text analysis when the eligibility were met a (second phase). Documents not meeting the eligibility criteria were excluded. An initial pilot test was performed in the first phase by reviewers until a sufficient level of agreement was reached (inter-reviewer agreement and computing sensibility and sensitivity are presented in Additional file 3). After that, the remaining publications were divided between the reviewers. Two researchers independently reviewed the selected documents in the second phase. The reviewers resolved any discrepancies through consensus; whenever questions or doubts occurred, a third referee reviewer was consulted. The inclusion process is described in Fig. 1 as recommended by PRISMA [23].

### Data extraction

Data from selected documents were collected and managed using a form developed in the REDCap (Research Electronic Data Capture) tools [24, 25]. Selected documents were randomly distributed between the reviewers. The information extracted from the documents selected included bibliographic information (author, year, language), objective, study design, interventions (type, country, context, other relevant characteristics), HCWs and outcomes (when presented).

### Assessment of the risk of bias and certainty of evidence

Risk of bias was assessed with the Joanna Briggs Institute (JBI) Critical Appraisal Tools (CAT) [26], categorizing the documents as low, medium or high quality. The quality of the evidence supporting study findings regarding the outcomes of the policy and management interventions implemented by countries was analyzed using the approach developed by Grading of Recommendations, Assessment, Development and Evaluation (GRADE Pro) Working Group [27].

### Data synthesis

Narrative synthesis was used to review and synthesize the data extracted. Documents were summarized and categorized based on the WHO’s interim guidance on HCWF policy and management in the context of COVID-19 [14] (Table 2).

**Table 2:** Domains and areas of interventions presented at the interim guidance on HCWF policy and management in the context of COVID-19

Domains	Areas of interventions
Supporting and protecting HCWs (individual level)	Infection prevention and control*
	Decent working conditions
	Mental health of HCWs*
	Remuneration and incentives
Strengthening and optimizing HCWF teams (management level)	Building competencies through education and training
	Optimizing roles
	Leveraging community-based HCWs
Increasing capacity and strategic HCWs deployment (organizational level)	Improving health worker availability
	Rationalizing the HCWF distribution
	Supportive work environment and manageable workload
System-level HRH interventions (systemwide level)	Strengthening governance and intersectoral collaboration mechanisms
	Improving HCWF information systems
	Assessment, planning of HCWF needs
	Licensing and regulation

*HCWs* health and care workers, *HRH* human resources for health

Source: WHO (2021) [14]

*These areas of interventions were considered as part of the “Decent working conditions”

## Results

A total of 3378 records were identified: 69 were selected and included in the review (Fig. 1 and Additional file 4, excluded records available in Additional file 5).

### Documents characteristics

Of the 69 documents included in the review, 52 were published in 2021 and 66 were written in English. They were classified as text and opinion (*n* = 50—which included technical and political documents), followed by qualitative (*n* = 9), cross-sectional (*n* = 6), quasi-experimental (*n* = 3) or cohort studies (*n* = 1). Most of the records were assessed as medium or high quality (54% and 39%, respectively) for risk of bias (JBI’s CAT).

**Fig. 2:**
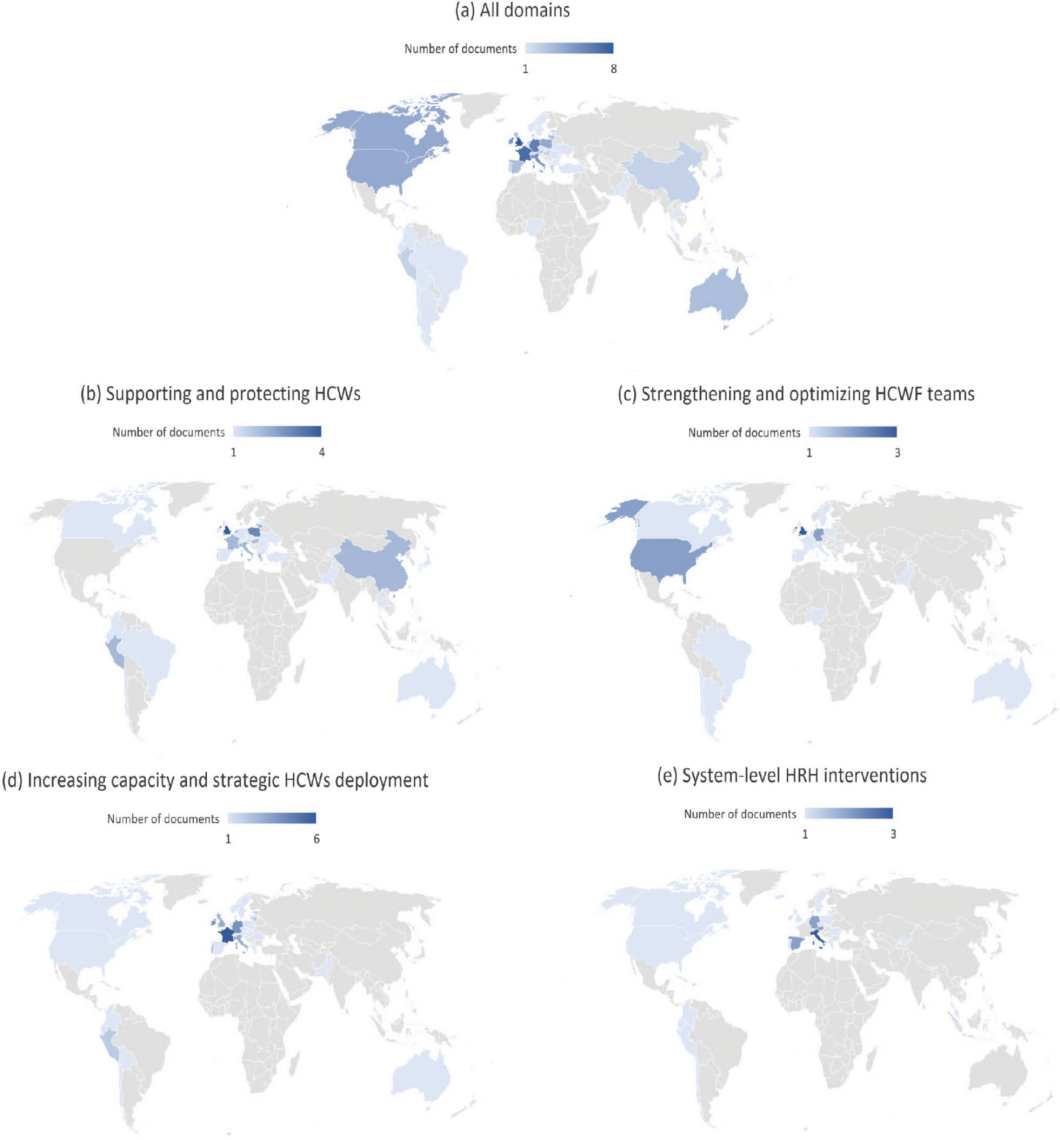
Geographical distribution of the studies included in the systematic review according to domain. Based on WHO’s [37] classification of country regions and the World Bank’s [38] country income levels

**Fig. 3:**
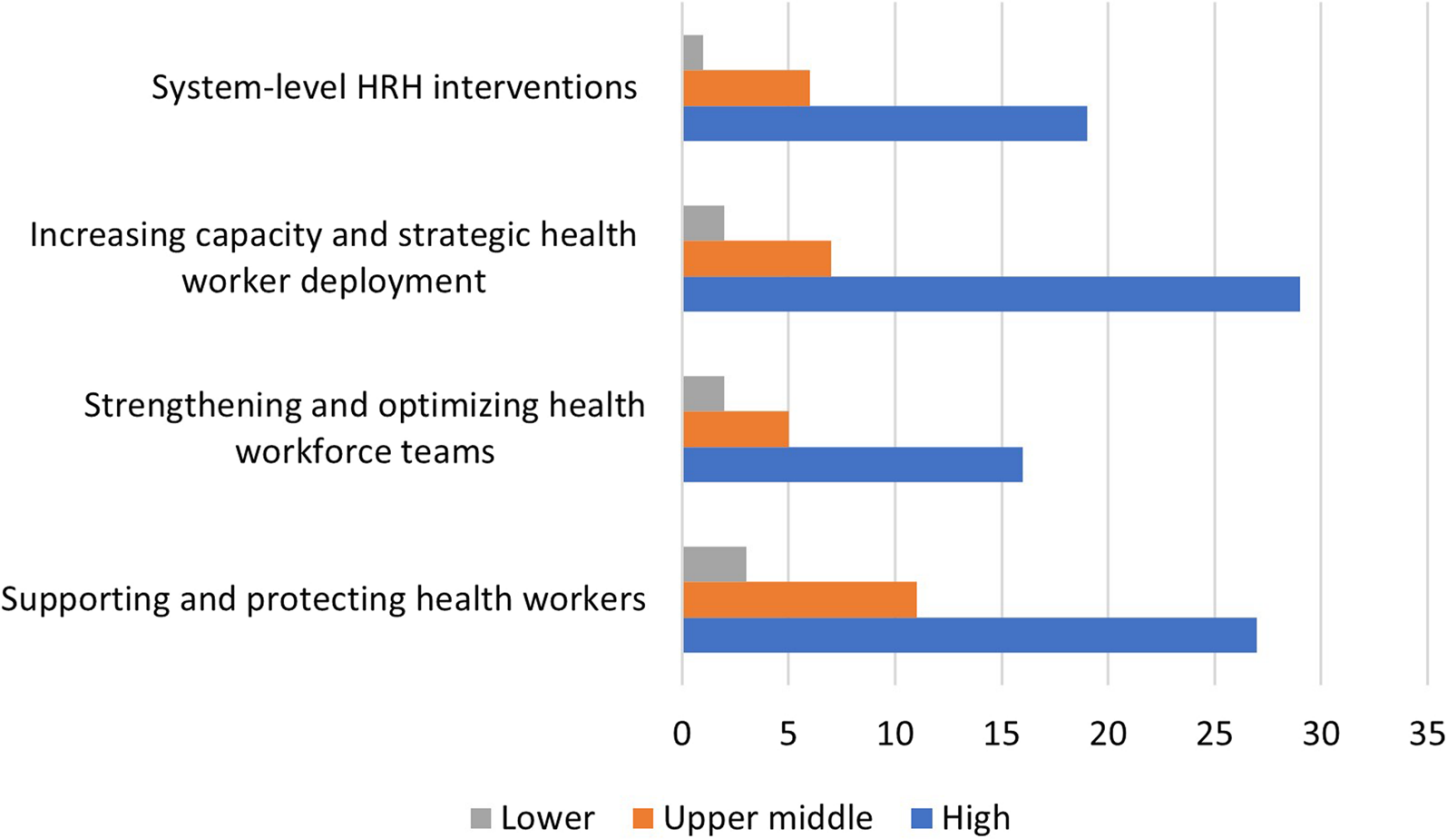
Distribution of countries covered in the documents reviewed by income level per domain. The World Bank [38] classification was used to group countries into income levels

The documents presented interventions implemented in 53 countries across six regions. Almost half of them (49%) were from high-income countries (HIC), while fewer came from lower-middle-income countries (LMIC) (7.2%) (Figs. 2a, 3). This distribution is maintained across all domains, with a large proportion of documents displaying interventions implemented in HIC and a smaller proportion of interventions in LMIC (Figs. 2 b, c, d, e, 3).

Interventions targeted different occupations, most addressing unspecified HCWs (*n* = 46), physicians (*n* = 34) and nurses (*n* = 29) (some documents may address more than one HCW category, see Additional file 4).

The availability of sex-disaggregated data was identified in nine documents, as part of the characterization of the population studied [28–33], addressing the (possible) gender implications of interventions [34, 35], or indicating of policies with a gender perspective/gender-sensitive approach, but without specifying how this was implemented [36].

### Policies and management interventions implemented by countries

The majority of the literature identified and reviewed presented at least one intervention implemented by countries that were classified under the domain “Increasing capacity and strategic HCWs deployment” (*n* = 44) followed by “Supporting and protecting HCWs” (*n* = 37) (documents may provide interventions in different domains, Table 3).

**Table 3:** Summary of the policy and management interventions identified by domain and area of intervention

Domains	Areas of interventions	Interventions
Supporting and protecting HCWs (individual level)	Decent working conditions	• Programs of training for use of PPE, biosafety measures and infection prevent control [30, 39, 42, 43, 46] • Establishment epidemiological monitoring and infection control, harmonization of standard operating procedures [41, 45] • Policies to access to PPE [34, 44] • Reassignment of HCWs at high risk [40, 47] • Campaign to reduce harassment and violence against HCWs [48] • Provide mental health and well-being [33, 56–58, 60, 97] • Vaccination for HCW as priority groups [44, 49–51, 53, 54] and mandatory for HCWs [52]
	Remuneration and incentives	• Increase resources and budget reallocations to HCWs [52, 62] • Financial incentives, such as salary adjustment, extra hours, special bonuses and others [42, 44, 53, 54, 58, 63–71] • Non-financial incentives, such as: free accommodation and transportation, scholar credits, support for children by organized childcare or provide a bonus for the purchase of babysitting services and others [43, 58, 59, 65, 72] • Financial compensation, such as paid leave, insurance cover and others [33, 34, 68, 73]
Strengthening and optimizing HCWF teams (management)	Building competencies through education and training	• Online training, such as online course, platforms, mobile applications and others [28, 32, 35, 74–76] • Supervision of online specialists for immediate consultation by professionals in specific care situations [36] • Supervision assurance to reinforce skills acquired to provide care beyond usual professional skills [98] • Support from professional associations in training the HCWF [39] • Review of national guidelines and development of training according to current evidence [40, 45, 77]
	Optimizing roles	• Expansion of scope of practice [42, 59, 70, 81] • Shift in responsibilities and relocation to face shortage skills [54, 78, 80, 98] • Availability of personnel for dedicated hotlines, apps and telemedicine [34, 43] • Redistribution of tasks among HCWF to take advantage of scope of available skills [34, 69, 71]
	Leveraging community-based HCWs	Not found
Increasing capacity and strategic HCWs deployment (organizational)	Improving HCWs availability	• Strategies to improve availability, such as volunteering, freelance, short term and temporary contract [41, 47, 76] • Redeployment such as relocated workers from other sectors and from private or public sector [88] Mobilization of non-health workers to perform no medical support tasks in areas, where additional workforce was needed, and mobilization of teachers, academics, retired professional [42, 44, 47, 49, 51, 52, 54, 55, 59, 62–65, 69, 73, 75, 76, 80–87] • Temporary licenses or hiring without validation of qualification for overseas trained professionals [42, 85] • • Asking to work extra hours (e.g., expand shift lengths, part time to work full time), cancelling leaves of absence or planned retirements of existing personnel and prohibiting workers from leaving the country to increase the capacity of the existing health workforce [47, 52, 53, 59, 63, 80, 85]
	Rationalizing the HCWF distribution	• Temporary redeployment of experienced staff [31, 42, 55, 88, 89] • Range of strategies to expand the use of telehealth [29, 34, 39, 43, 44, 51, 52, 54, 55, 59, 62, 66, 67, 69, 70, 77, 79, 80, 83, 84, 86, 87, 90–92]
	Supportive work environment and manageable workload	• Strengthen existing or institute a supportive mechanism for better communication, such as call center and ethical support units [48, 93]
System-level HRH interventions (systemwide)	Strengthening governance and intersectoral collaboration mechanisms	Fast track legislation and intersectoral collaborations to increase availability [47, 52, 55, 73, 86] Financial strategies to respond to COVID-19 such as allocation of financial resources, recovery plan and adjustment to ensure availability of funds to pay for COVID-19 services [42, 54, 59, 66, 69, 79, 81, 82]
	Improving HCWF information systems	• Health information system to planning tools to rapidly assess workforce requirements such as monitoring reporting absence system [60] • Implementation of database in nursing homes for monitoring PPE and professional shortage [94]
	Assessment, planning of HCWF needs	• Creation of database of inactive workers; health service reserve list; mandatory census of all licensed health care practitioners [43, 44, 50] • Temporary suspension of regulations [80]
	Licensing and regulation	• Short-term training to professionals from abroad; waive of administrative process requirement [42, 73, 76] • Measures to loosen regulations [34, 41, 42, 65, 66, 73, 76, 79, 86] • Recognition of foreign training and accelerated licensing or credentialing [42, 72, 73, 76, 95, 96]

**Fig. 4:**
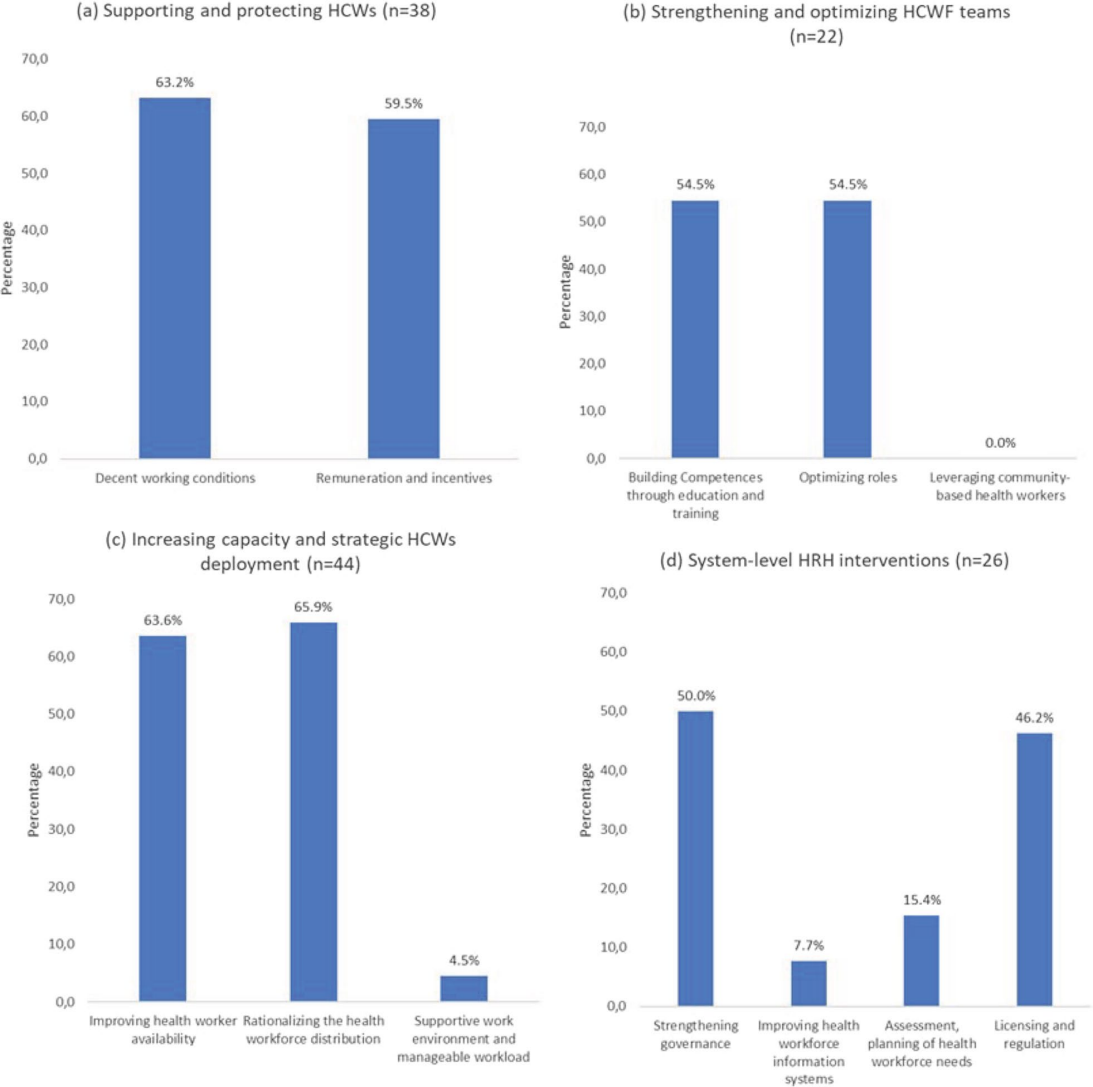
Percentage of documents included in the review that address each domain by intervention area. Documents may contain interventions implemented in more than one country, in more than one domain and/or in more than one area of interventions

Regarding the area of intervention, most documents presented interventions implemented in more than one area (*n* = 45). Those that presented interventions implemented in a single area were equally distributed in “decent working conditions” (*n* = 6) and “rationalizing the HCWF distribution” (*n* = 6). Observing the distribution of documents according to the area of intervention within each domain, it was possible to verify that the area “supportive work environment and manageable workload” was the least documented (only 2 of 44 documents addressed this domain). In addition, there was a lack of studies and reports on what was being done by countries in “leveraging community-based health workers” (Fig. 4, items a, b, c and d, Table 3).

### Supporting and protecting health and care workers (individual level)

A significant portion of the literature identified and reviewed presented policy and management interventions put in place by countries at individual level with the intention of attracting and retaining HCWs in a safe and decent working environment (*n* = 38). These documents cover 41 countries across five regions (except Africa) and target different occupations, most of them addressing unspecified HCWs (*n* = 34) and physicians (*n* = 27), referring mostly to interventions in the area of “decent working conditions” (*n* = 24).

In this area, “decent working conditions”, actions implemented to protect HCWs, included training sessions on infection prevention and control, developing guidance on the use of PPE and implementing epidemiological COVID-19 surveillance [30, 32, 34, 39–45]. HCWs at high risk were sometimes reassigned [40, 46, 47]. One publication describes a campaign to reduce discrimination against HCWs [48]. HCWs were always included among the priority groups for vaccination and mobile units to support on-site vaccination were used to ensure accessibility [44, 49–55]. Furthermore, to support mental health and well-being of HCWs, countries provided psychological helplines [33, 56, 57], developed databases of mental health specialists willing to offer their services free of charge [58, 59], created counselling programs [60], offered free mental health support [58] and established single-point-of-access resources to which HCWs could turn for advice [61].

Regarding “remuneration and incentives”, countries increased resources and reallocated budget to ensure payment of salaries and bonusses [52, 62] and employed financial incentives to attract HCWs to act in response to COVID-19, to compensate for higher COVID-19 workloads and risks and to reduce attrition [42, 44, 53, 54, 58, 63–71]. Non-financial incentives such as (temporary) accommodation expenses and continuing education credits were also used, on their own [43, 58, 65, 72] or in combination with financial incentives [58, 59, 73]. Countries also paid for social protection provisions, such as incentives to self-isolate, doubling of financial compensation to COVID-19-positive HCWs to take leave to care for an ill elderly persons, and provided insurance to cover cases of disability or death due to COVID-19 or related sickness [33, 34, 68].

### Strengthening and optimizing health and care workforce teams (management level)

Strategies adopted by countries in this domain were described by 22 documents covering 23 countries in five regions (except for South–East Asian Region). Unspecified HCWs were the most cited (*n* = 13), followed by physicians and nurses, both with 10 documents.

Interventions that were developed to “build competences through education and training” were described in 12 of the documents addressing this domain. Countries used national and global platforms to fast-track training and accreditation of specific skills through e-learning tools [28, 35, 74–76]. For example, in Nigeria, the use of the InStrat COVID-19 app allowed HCWs to access COVID-19 training modules [28]. The National Institute of Health in Italy made efforts for quality improvement in the use of digital tools aimed at training and developing the skills of HCWs through distance learning, by creating dedicated webinars and distance courses, associated with Continuing Medical Education credits [77].

The literature revealed strategies to ensure that HCWs received adequate guidance, training and supervision to safely deliver care that extended beyond their competencies and skills. For example, in Argentina, due to a shortage of trained critical care HCWs, the Ministry of Health's National Directorate of Talent and Human Knowledge (DNTHyC) implemented the Localized and Permanent Training Contingency Project to train and assist HCWs who cared for hospitalized COVID-19 patients in critical condition. The Tele-Revista tool was used to supervise intensive care units (ICU) across the country, and online consultations with specialists were made available to help HCWs in specific situations [36].

For “optimizing roles”, measures regarding task sharing, task transfer, expansion of responsibilities, HCW collaboration, configuration of teams and allocation of students to meet population clinical needs were implemented, with physicians being the most cited profession (*n* = 5), followed by nurses (*n* = 4). Interventions included: revisions in scope of practice with the performance of tasks normally assigned to physicians by qualified nurses and emergency paramedics [78]; broadening the scope of practice of some HCWs during the pandemic; and allowing caregivers with shorter or no training to provide health care usually restricted to health professionals [79]; pharmacists were allowed to provide medicines to patients without a prescription as long as the drugs were part of an ongoing treatment [78]; authorities mobilized students of medicine, nursing and health sciences to work in clinical practice, to provide assistance with COVID-19 pandemic counseling hotlines, as well as contact tracing [80].

Furthermore, to ensure the provision and maintenance of health care and to improve responsiveness, several team configuration were employed by countries, such as the National Vaccination Plan in Chile, which, in addition to having nursing teams, included other HCWs, such as midwives and licensed dentists [70]; Poland, seeking to accelerate the implementation of vaccination against COVID-19, authorized physiotherapists, pharmacists and laboratory diagnosticians, after relevant training, to administer the vaccines [59]. In addition, nurses were reallocated from other sectors of the hospital to work together and under the supervision of nurses with experience in the ICU [69]. Dentists, especially those with skills in sedation, were also reallocated to support National health Services´ (NHS) hospital care during the pandemic [71].

### Increasing capacity and strategic health and care worker deployment (organizational level)

Most of the interventions reviewed focused on this domain (*n* = 44 documents describing at least one intervention), covering 38 countries across five regions (The Americas, South–East Asia, Europe, Eastern Mediterranean and the West Pacific) and targeting various occupations; mostly unspecified HCWs (*n* = 45), physicians (*n* = 28), and nurses (*n* = 25). These documents predominantly refer to interventions in the area of “rationalizing the HCWF distribution” (*n* = 29).

In the area of “improving HCWs availability”, interventions, such as recruitment within and outside the health and care sector, reassigning workers and taking steps to bring in retired or inactive HCWs, students and overseas trained HCWs were implemented [42, 44, 47, 49, 51, 52, 54, 55, 59, 62–65, 69, 73, 75, 76, 80–87]. In addition, countries increased the capacity of the existing HCWF by asking staff to work extra hours and cancelling leaves of absence or planned retirements [47, 52, 53, 59, 63, 80, 85]. Furthermore, volunteers from the general public and/or non-HCWs were also mobilized to play different roles [41, 47, 76]. In addition to hiring extra HCWs using different types of contracts (volunteer, freelance, short-term, temporary and permanent), undergraduate students and HCWs in specialist training (specialties or subspecialities) [83] served as resources for support roles [49] and to provide care to COVID-19 patients [63, 75], as part of a reserve list [76] or in helplines [64, 80], rapid response, case investigation, contact tracing [41, 80], and psycho-social support [41]. Many HCWs were incorporated into the health labor market as pre-existing requirements to practice were suspended temporarily [42], consequently allowing them to be recruited [42, 82, 85].

Under the intervention area of “rationalizing HCWF distribution”, countries (temporarily) redeployed experienced staff from low-to-high-burden settings and to areas of greater need to respond to a massive influx of patients, avoiding disruption of essential services and maintaining population access [31, 42, 55, 88, 89]. There was also a rapid scale-up of remote delivery of care by digital health tools, where teleconsultation played an essential role [29, 34, 39, 43, 44, 51, 52, 54, 55, 59, 62, 66, 67, 69, 70, 77, 79, 80, 83, 84, 86, 87, 90–92]. Digital tools were employed to support the provision of COVID-19-related care, such as remote monitoring of COVID-19 patients in isolation at home [70, 84], and carrying out remote medical triage to issue or renew prescriptions and referrals by phone or video consultations [70, 90]. In addition, to meet needs unrelated to COVID-19, such as maintaining access to ambulatory care [83] and chronic conditions [54], connecting long-term care facilities with geriatric specialists [34] and extending the remote delivery of primary health care services provided by a multi-profile team comprising complex health needs [65].

Lastly, two documents addressed the area of intervention “supportive work environment and manageable workload” to strengthen existing or institute regular supportive supervision mechanisms for better communication and support of HCWs by implementing a call center for effective communication [93] and developing ethical support units [48].

### System-level interventions (systemwide level)

System-level interventions adopted by countries were described by 26 documents, covering 26 countries across three regions (The Americas, South–East Asia, Europe), mostly in European countries. The literature presented interventions targeting different types of occupations, mostly unspecified HCWs (*n* = 24), physicians (*n* = 15), and nurses (*n* = 13).

Half of the documents analyzed described strategies in the area of "Strengthening governance and intersectoral collaboration mechanisms" presenting interventions related to financial incentives to encourage hiring, purchase of materials and development of plans to enable the continuity of these measures, such as allocation of financial resources to develop integrated care services, recovery and resilience plans, and legislative budget adjustments to ensure the availability of funds needed to pay for COVID-19 services [42, 54, 59, 66, 69, 79, 81, 82].

To allow fast-track legislation, and intersectoral collaborations to increase the availability of HCWs, by changing the recruiting, planning, and integration of these new workers into clinical practice, it was necessary to draft emergency legislation to grant health care planners, providers, and commissioners additional temporary powers [47, 52, 55, 73, 86].

High-level diplomacy between countries and coordination between sectors, for example in Malaysia, government organizations made available staff members from the Ministries of Defense, Housing, Human Resources, and Local Government to help public health teams through the collaboration of nongovernmental organizations [47].

Only two documents addressed the topic of "Strengthening human resources for health information systems," with the interventions primarily consisting of the creation or adaptation of an existing database to monitor PPE and HCWs’ shortages [94]; the use of available HCWF data collected both nationally and locally, as well as the number of patients hospitalized over time and a weekly updated absence monitoring and reporting system, with absence rates reported (COVID or non-COVID related) [60].

Strategies such as compiling a list of retired HCWs and enlisting volunteers to work on a temporary basis [44, 50], developing a platform for both medical and non-medical volunteers [43], and temporarily suspending regulations that required a certain number of nursing professionals to work in intensive care or geriatric units [80] were identified in the area of “assessment and planning”.

For “licensing and regulation” countries loosened regulations for practicing and licensing [42, 65, 66, 72, 73, 76, 79, 86, 95], suspended mandatory enrollment in the professional register [34] and eased the recognition of degrees of foreign doctors [96]. In the Maldives, due to the lack of qualified HCWs the government implemented policies to make it easier for Maldivian students abroad to return, and to scale back regular health services, so that existing HCWs could be released for the COVID-19 response [41].

### Effectiveness of policies and management interventions

Despite the fact that the documents identified in this review contained a wide range of policy and management interventions used by countries, only a small proportion of them presented outcomes [28–32, 35, 45, 63]. In addition, the reviewed literature did not review any outcomes of measures adopted by the countries on a system-wide level. Furthermore, there is not enough data to draw conclusions on the impact of these interventions to improve HCWs accessibility and availability, as only one study provided general information on the effect of the intervention of additional temporary hiring of health personnel on HCWs availability [63]. The evidence presented for these outcomes was mainly obtained through observational studies and classified, according to GRADE Pro [27], as mostly low-certainty evidence (Table 4).

**Table 4:** Outcomes, described for the implementation of the policy and management interventions and quality of evidence

Area of intervention	Outcome	Brief description	Overall quality
Decent working conditions	Protection of HCWs against infection	*Training on use of PPE, instructed to practice social distance and hotels with only designated for medical staff* 943 health professionals from Guangzhou that were sent to assist Wuhan to combat COVID-19, tested negative for all four reverse transcription polymerase chain reaction (RT-PCR) performed on days 1, 2, 7, and 14. The local healthcare workers in Wuhan and Jingzhou, 2.5% (113 out of 4495) and 0.32% (10 out of 3091) had RT-PCR confirmed COVID-19, respectively. The seropositivity for SARS-CoV-2 antibodies (IgG, IgM, or both IgG/IgM positive) was 3.4% (53/1571) in local healthcare workers from Wuhan with Level 2/3 PPE working in isolation areas and 5.4% (126/2336) in healthcare staff with Level 1 PPE working in non-isolation medical areas, respectively [30] *Intensification of COVID-19 epidemiological surveillance, distance learning seminars (continuous education), communication, feedback to the Heads of the long-term care facility (LTCF), harmonization of Standard operating procedures and intensification of audits to the LTCF, promotion of volunteerism and active participation of medical students, and task force activation on confirmed case identification and cluster events* The results indicated a statistically significant decrease in COVID-19 cases between the first and second decade of December 2020 for Cyprus LTCF. During the interventional period, a significant decrease of 47% in COVID-19 cases was observed in the LTCFs (reduction of the prevalence from 2.83% to 1.5%). The results indicated a statistically significant decrease in COVID-19 cases (χ2 = 19.42, p < .001) between the first and second decade of December 2020 for Cyprus LTCF Total (from 138/4878; 2.83% 95%CI [2.40% − 3.33%] to 71/4740; 1.5% 95%CI [1.19% − 1.89%]), as well as a significant decrease (χ2 = 19.29, p < .001) for Cyprus LTCF Residents (from 107/ 2928; 3.65% 95%CI [3.03% − 4.40%] to 49/2817; 1.74% 95%CI [1.31% − 2.30%]) but a non-statistically significant difference (χ2 = 1.41, p = .24) for Cyprus LTCF Staff (from 31/1950; 1.59% 95%CI [1.11% − 2.26%] to 22/1923; 1.14% 95%CI [0.75% − 1.74%]) [45]	Low
Decent working conditions	Improved knowledge	*Training of biosecurity measures for nurses exposed to SARS-CoV-2 in emergency sectors* An educational intervention (10 modules—318 h) with 80 nurses (26 technicians, and 54 graduates), duration of 5 weeks. Before intervention both groups had insufficient knowledge regarding COVID-19, after intervention the level of knowledge of COVID-19, standards of biosafety increased in both groups. The educational intervention was effective with statistical significance in the level of knowledge of the group licensed regarding the technician. The level of knowledge of COVID-19 rose after the intervention (69,23% group I, 74.07% group II), while the knowledge on principles and standards of biosafety increased in both groups (88.46% and 100%). The knowledge about precautions standards rose in 65.38% technical group and 92.59% graduates’ group. Group I (26 technicians) and group II (54 graduates)[32]	Low
Building Competences Through Education and Training	Improved knowledge	*Nationwide electronic learning (e-Learning) intervention was implemented across 25 states of Nigeria, using a tutorial app with 7 training modules, consisting of video, audio and text-based learning materials, available in English and then translated to three major languages: Hausa, Igbo and Yoruba* A total of 1051 health workers from 25 states across Nigeria undertook the e-learning on the InStrat COVID-19 training app. Of these, 627 (57%) completed both the pre- and post-tests in addition to completing the training modules. Overall, there were statistically significant differences between pre- and post-tests knowledge scores (54 increasing to 74). There were also differences in the subcategories of sex, region, and cadre. There were higher post-test scores in males compared with females, younger versus older and southern compared with northern Nigeria. A total of 65 (50%) of the participants reported that the app increased their understanding of COVID-19, while 69 (53%) stated that they had applied the knowledge and skills learnt at work. Overall, the functionality and usability of the app were satisfactory [28] *A 5-week online training program for healthcare professionals on prevention and control of SARS-CoV-2 infection. The objective knowledge assessment was carried out using a total of 110 test questions, with four response options. The participants had to pass each test with at least 80% correct answers* Of the 880 healthcare professionals pre-enrolled on the course, 766 (87.1%) started the training. From these, 705 (92.0%) success fully passed assessments and completed the pre-and surveys (represents 29.12% of the total number of healthcare professionals in Tenerife). The pre-training median total score of perceived knowledge score was 40 (29–53) points, which the post training total score was 53 (39–60) points, confirming significance in this difference (p < 0.001, Wilcoxon’s Z: –22.407). The results of this study suggest a high level of self-perceived knowledge acquired in all areas assessed and related to the prevention and control of SARS-CoV-2 infection in healthcare professionals who completed the training program [35]	Low
Improving HCWs availability	Health workforce availability	*MINSA (Ministry of Health) and regional government facility staffing per subsector from additional temporary hiring of health personnel hires, the additional contract workers were utilised in Rapid Response Teams* Increase in MINSA and regional government facility staffing per subsector. In response to COVID‐19 there was an additional contract in 10,44%, a total of 26,120 additional contracts, with 4640 medical, 6467 nurses,1272 midwifes, 8325 technical assistants and others [63]	Very Low
Rationalizing HCWF	Protection and personal well-being (burnout)	*Nationwide cross-sectional survey was design to understand the impact of COVID-19 pandemic on junior and middle grade doctors working for National Health System in the United Kingdom* Out of 1564 (survey questionnaire) respondent 61.6% of doctors were redeployed outside their primary specialty. The major redeployments were from other specialties to intensive therapy unit (ITU)/critical care units (CCU) (41.8%). This was secondary to expansion in critical care capacity across all hospitals particularly in tertiary care hospitals. The majority of deployments were from medicine and allied specialties (54.4%); 63.3% of respondents spend more than 8 weeks in redeployed specialty with majority of doctors from medicine followed by anesthesiology. In general, anesthesiology and medicine and allied were more significantly affected specialties by this mass redeployment. When burnout was gauged using single questions with the highest factor loading on the EE and DP, 85.25% (*n* = 1333) and 64.7% (*n* = 1012) responded positively, suggesting very high impact of COVID-19 on doctors’ well-being [31]	Low
	Impact on clinical work (working conditions)	*Nationwide cross-sectional survey was design to understand the impact of COVID-19 pandemic on junior and middle grade doctors working for National Health System in the United Kingdom* Majority of doctors had an impact of COVID-19 on their clinical practices irrespective of the fact if they stayed in their primary specialty or redeployment elsewhere. This all happened due to unfamiliar surroundings, increased work demand, nature of COVID-19 disease causing sudden deterioration of the patients, and rapid influx of patients to hospitals. This unprecedented work intensity required more support for junior and middle grade doctors, which unfortunately was not readily available that resulted in more adverse impact on physical and mental well-being of these doctors. Various areas for improvement were suggested. The major areas requiring immediate attention include proper leadership and clinical support (64.1%), pre redeployment planning and induction (48.5%), redeployment according to the skills and/or in familiar specialties (44.6%), and regular mental and physical well-being checks (37%) [31]	Low
	Professional's satisfaction	*National Health System Portugal. The hospital administrations and services, and the Ministry of Health, preferably recommended the teleconsultation activity, reserving face-to-face consultations for when teleconsultation was not clinically adequate or technically possible* A total of 2452 answers were obtained, and 2225 answers were considered for analysis. The answers of doctors who were not working in the National Health System in the first phase of the pandemic were excluded. Thus, around 7.2% of doctors who worked in the National Health System responded to the questionnaire. 50% refer that they are globally satisfied or very satisfied with teleconsultation, 16% are dissatisfied or very dissatisfied and 35% are indifferent [29]	Low

**Note:** The body of evidence from observational research is initially categorized as low-quality evidence using the GRADE system and it was assessed whether the studies had limitations (risk of bias) that were serious enough to downgrade the quality of the evidence for this outcome

## Discussion and conclusions

The pandemic accentuated workforce issues in several countries when politicians and managers had to make decisions quickly to face the pandemic. Thus, the traditional planning process had to be accelerated and measures taken. At the same time, the pandemic also impacted the publication process with the need for rapid dissemination of information, more documents concerned with describing interventions and less with evaluating them.

Most of the interventions identified were in the European region followed by the Americas. Consequently, the results may not be generalizable to other regions. In addition, the documents examined in this review, which were mostly observational and technical and policy documents, not allowing for the evaluation of the results, and most were classified as low-quality evidence.

While the reviewed documents do not explicitly address gender issues, some of the interventions implemented address key points of the topic's challenges, such as the expansion of working hours for part-time workers, which may have had a greater direct impact on women, because they are more likely to work part-time [99], also the availability and use of non-financial benefits, such as childcare support, given the increase in care responsibilities at home when schools and childcare support were restricted [10]. The limited availability of sex-disaggregated data has been identified as a challenge, limiting the analysis of gender implications associated with the outcomes of implementing interventions and policies in the context of COVID-19. Hence, to advance equitable and inclusive strategies, it is crucial to prioritize and integrate gender analysis and perspectives in future studies related to HCWF.

The evidence regarding the interventions implemented by countries is still weak; therefore, the impact and efficacy of the strategies used by countries during the pandemic still require further research, and it is crucial that the measures taken by policymakers are long-lasting and sufficient to ensure the viability of the workforce and enable working conditions that are appropriate for HCWs.

Many of the systemwide HCWF enablers were viewed as intervention facilitators, with little research into how they were (re)structured and adapted to allow for agility in implementation. Furthermore, the results described are influenced by other factors, so it cannot be said that they are exclusively related to the strategies described, highlighting the need to promote high-quality methodological studies on the subject and the inclusion of robust evaluations to determine the effectiveness of the described strategies and better inform the HCW policy-making process.

### Supplementary Information

**Additional file 1.** Search tool specification and corresponding terms.

**Additional file 2.** Search strategies.

**Additional file 3.** Inter-reviewer agreement and sensitivity.

**Additional file 4.** Details on documents included in the review.

**Additional file 5.** Tables of excluded documents.

## Data Availability

The database of the literature review is available upon request from the authors.

## Abbreviations

BVS: Virtual Health Library
CAT: Critical appraisal tool
COVID-19: Coronavirus Disease 19
GRADE: Grading of Recommendations, Assessment, Development, and Evaluations
HCS: Health and care systems
HCWF: Health and care workforce
HCWs: Health and care workers
HIC: High-income countries
HRH: Human resources for heath
HSRM: The Health System Response Monitor
ICU: Intensive Care Unit
ILO: International Labour Organization
JBI: Joanna Briggs Institute
LILACS: Latin American and Caribbean Health Sciences Literature
LTCF: Long-term care facilities
LMIC: Lower-middle-income countries
NHS: National Health Service
OECD: Organization for Economic Co-operation and Development
PPE: Personal Protective Equipment
PUBMED: US National Library of Medicine National Institutes of Health
SARS-CoV-2: Severe Acute Respiratory Syndrome Coronavirus 2
WHO: World Health Organization
