# Supplementary material for: Implementation of policy and management interventions to improve health and care workforce capacity to address the COVID-19 pandemic response: a systematic review
Source: Hum Resour Health. 2023 Oct 10;21:80. doi: 10.1186/s12960-023-00856-y (PMC10563305; doi:10.1186/s12960-023-00856-y)
Supplement: Supplementary file 1 — Additional file 1. Search tool specification and corresponding terms. [file 12960_2023_856_MOESM1_ESM.docx]

**Additional file 1 – Search Tool Specification and corresponding terms**

| **PICOC** | **Description** | **Specification** | **Terms to search** |
| --- | --- | --- | --- |
| Population | Heath care workers | Health workforce | Human resources for health, health professionals, health personnel, health workforce, health manpower, health and care workers, health and care workforce, health occupations, health care provider, healthcare provider, healthcare worker, health care worker, health care professional, healthcare professional, caregivers  Dentist, doctor, physician, nurse (licensed practical nurses, nursing staff, nursing personnel, nursing staff, professional nurse or nursing associate, nursing professional, nursing assistant, auxiliary nurse, nursing auxiliary, licensed practical nurse, nursing team), pharmacist, physiotherapist, midwife, community health workers, community-based providers, paramedical staff, laboratory staff |
| Intervention | Policy and management interventions aiming to improve health workforce capacity to address the covid-19 pandemic response | Policy and management interventions | health policy, health planning, interventions, strategies, measure, coping, shortage, pandemic response |
| **C**omparison | no intervention or other intervention (no document/study will be excluded irrespective of the comparisons reported) | No search terms will be used to limit the search to documents that compare interventions. Documents will not be excluded, regardless of the comparisons reported. |  |
| **O**utcome | Improvement of health workforce capacity to address the COVID-19 pandemic response | No search terms will be used to limit the search to documents that show results of the implemented interventions. Documents will not be excluded, regardless of the results reported. |  |
| **C**ontext | Covid-19 pandemic response | COVID-19 OR SARS-CoV-2 | COVID-19 Virus Disease, COVID-19 Virus Infection, COVID 19 Virus Infection, 2019-nCoV Infection, 2019 nCoV Infection, Coronavirus Disease-19, Coronavirus Disease 19, 2019 Novel Coronavirus Disease, 2019 Novel Coronavirus Infection, 2019-nCoV Disease, 2019 nCoV Disease, COVID19, Coronavirus Disease 2019, Wuhan Coronavirus, SARS Coronavirus 2 Infection, SARS-CoV-2 Infection, SARS-CoV-2 Infection, COVID-19 Pandemic, COVID 19 Pandemic |
