# Supplementary material for: Implementation of policy and management interventions to improve health and care workforce capacity to address the COVID-19 pandemic response: a systematic review
Source: Hum Resour Health. 2023 Oct 10;21:80. doi: 10.1186/s12960-023-00856-y (PMC10563305; doi:10.1186/s12960-023-00856-y)
Supplement: Supplementary file 3 — Additional file 3. Inter-reviewer agreement and sensitivity. [file 12960_2023_856_MOESM3_ESM.docx]

**Additional file 3 –Inter-reviewer agreement and sensitivity was computed to assess**

Inter-reviewer agreement was measured by estimating Kappa statistics and computing sensibility and sensitivity was computed to assess and the quality of this process using the SPSS software. The Kappa result be interpreted as follows: values ≤ 0 as indicating no agreement and 0.01–0.20 as none to slight, 0.21–0.40 as fair, 0.41– 0.60 as moderate, 0.61–0.80 as substantial, and 0.81–1.00 as almost perfect agreement ^[[1]](#footnote-1)^. The kappa value was calculated for the first 200 articles for phase 1, the reviewers were divided into pairs K1 (LSM and MLG) and K2 (APCO and MLG) the results for the two independent reviewers were 0,823 and 0,771, considered almost perfect and substantial respectively. For the sensibility and sensitivity was 97,1% and 80% for K1 and 97,8% and 100% for K2.

1. McHugh ML. Interrater reliability: the kappa statistic. Biochem medica 2012; 22: 276–282. [↑](#footnote-ref-1)
