# Supplementary material for: Implementation of policy and management interventions to improve health and care workforce capacity to address the COVID-19 pandemic response: a systematic review
Source: Hum Resour Health. 2023 Oct 10;21:80. doi: 10.1186/s12960-023-00856-y (PMC10563305; doi:10.1186/s12960-023-00856-y)
Supplement: Supplementary file 4 — Additional file 4. Details on documents included in the review. [file 12960_2023_856_MOESM4_ESM.docx]

**Additional file 4** **- Details on documents included in the review**

| **Author and Year** | **Objective** | **Which question answers** | **Type of publication** | **Language** | **Country** | **Area of intervention** | **Study design** | **Profession (study population)** | **Critical Appraisal (Assessment of risk of bias)** |
| --- | --- | --- | --- | --- | --- | --- | --- | --- | --- |
| Iqbal et al 2020 [39] | Evaluate the national response of the health system of Pakistan to the COVID-19 pandemic according to scientific parameters. | 1 | Article | English | Pakistan | Decent working conditions, Building Competences through education and training, and Rationalizing the HCWF distribution | Qualitative | Health and care workers not specified | Low |
| Leite et.al 2021 [40] | To analyze federal government interventions in crisis management and the consequences for health professionals. | 1 | Article | Portuguese | Brazil | Decent working conditions, and Building Competences through education and training | Qualitative | Health workers, physicians, dentist, nurses and pharmacist | High |
| Usman et al 2021 [41] | Explores the planning assumptions, resource estimations and strategies adopted to equip the health system with resources for the pandemic response. | 1 | Article | English | Maldives | Decent working conditions, Improving health worker availability and Licensing and regulation | Qualitative | Nurses, physicians and health and care workers not specified | Medium |
| Winkleman et al 2021 [78] | To analyses the strategies that 45 countries in Europe have taken to secure sufficient health care infrastructure and workforce capacities to tackle the crisis, focusing on the hospital sector. | 1 | Article | English | 45 countries (data extracted from Germany, France, Austria, England) | Optimizing roles | Cross-Sectional | Physician, nurse, pharmacist, biomedical analyst, paramedical, graduates of natural and veterinary sciences and caregivers | Medium |
| Köppen et al 2021 [85] | To analyze the planning and health workforce policies in Germany, a country with a largely decentralized workforce governance mechanism. | 1 | Article | English | Germany | Improving health worker availability | Qualitative | Health and care workers not specified | Low |
| Unruh et al 2021 [88] | To compare health policy responses to COVID-19 in Canada, Ireland, the United Kingdom and United States of America (US). | 1 | Article | English | Canada; Ireland, UK and United States of America | Improving health worker availability and Rationalizing the HCWF distribution | Cross-Sectional | Nurses, physicians and health and care workers not specified | High |
| O´leary et al 2021 [89] | To document the healthcare policies developed during the initial wave of widespread COVID-19 transmission in Ireland. | 1 | Article | English | Ireland | Rationalizing the HCWF distribution | Qualitative | Health and care workers not specified | Medium |
| Glatman-freedman et al 2020 [93] | To describe and analyze the operation of the call center established by the Israel Center of Disease Control - ICDC (which belongs to the Israel MOH) in order to facilitate effective communication with health care providers during the early stages of the COVID-19 public health emergency. | 1 | Article | English | Israel | Supportive work environment and manageable workload | Cross-Sectional | Health and care workers not specified | Medium |
| McGarry et al. 2020 [95] | As part of recent federal COVID-19 nursing home data collection efforts, the Centers for Medicare and Medicaid Services (CMS) has begun collecting data from nearly every nursing home in the country regarding COVID-19 and its impact on residents and staff. To report some of the first results from these federal data by describing nursing home access to PPE and staffing and examining the facility characteristics associated with shortages in these areas. | 1 | Article | English | United States of America | Improving health workforce information systems | Cross-Sectional | Nurses, physicians and other | High |
| Silberman et al. 2020 [36] | To describe the approach of the human talent policy in the emergency of COVID-19. | 1 | Policy and technical document | Spanish | Argentina | Building Competences through education and training | Text and opinion | Nurses, physicians and health and care workers not specified | High |
| Liseckiene 2021 [92] | To present the digital transforming primary health care during the pandemic. | 1 | Policy and technical document | English | Lithuania | Rationalizing the HCWF distribution | Text and opinion | Nurse, physicians, physiotherapist and health care workers not specified | Low |
| OECD 2021 – Ireland [91] | The State of Health in the EU’s Country Health Profiles provide a concise and policy-relevant overview of health and health systems in the EU/European Economic Area. The aim is to support policymakers and influencers with a means for mutual learning and voluntary exchange. | 1 | Policy and technical document | English | Ireland | Rationalizing the HCWF distribution | Text and opinion | Health and care workers not specified | Medium |
| OECD 2021 – Malta [90] | The State of Health in the EU’s Country Health Profiles provide a concise and policy-relevant overview of health and health systems in the EU/European Economic Area. The aim is to support policymakers and influencers with a means for mutual learning and voluntary exchange. | 1 | Policy and technical document | English | Malta | Rationalizing the HCWF distribution | Text and opinion | Nurses, physicians and physioterapist | Medium |
| OECD 2021 – Denmark [87] | The State of Health in the EU’s Country Health Profiles provide a concise and policy-relevant overview of health and health systems in the EU/European Economic Area. The aim is to support policymakers and influencers with a means for mutual learning and voluntary exchange. | 1 | Policy and technical document | English | Denmark | Improving health worker availability and Rationalizing the HCWF distribution | Text and opinion | Health and care workers not specified | Medium |
| OECD 2021 – Portugal [86] | The State of Health in the EU’s Country Health Profiles provide a concise and policy-relevant overview of health and health systems in the EU/European Economic Area. The aim is to support policymakers and influencers with a means for mutual learning and voluntary exchange. | 1 | Policy and technical document | English | Portugal | Improving health worker availability, Rationalizing the HCWF distribution, Strengthening governance and Licensing and regulation | Text and opinion | Health and care workers not specified | High |
| OECD 2021 – France [84] | The State of Health in the EU’s Country Health Profiles provide a concise and policy-relevant overview of health and health systems in the EU/European Economic Area. The aim is to support policymakers and influencers with a means for mutual learning and voluntary exchange. | 1 | Policy and technical document | English | France | Improving health worker availability and Rationalizing the HCWF distribution | Text and opinion | Medical and nursing students, inactive and retired health professionals | High |
| OECD 2021 – Norway [83] | The State of Health in the EU’s Country Health Profiles provide a concise and policy-relevant overview of health and health systems in the EU/European Economic Area. The aim is to support policymakers and influencers with a means for mutual learning and voluntary exchange. | 1 | Policy and technical document | English | Norway | Improving health worker availability and Rationalizing the HCWF distribution | Text and opinion | Health and care workers not specified | Medium |
| OECD 2021 – Italy [82] | The State of Health in the EU’s Country Health Profiles provide a concise and policy-relevant overview of health and health systems in the EU/European Economic Area. The aim is to support policymakers and influencers with a means for mutual learning and voluntary exchange. | 1 | Policy and technical document | English | Italy | Improving health worker availability and Strengthening governance | Text and opinion | Health and care workers not specified | High |
| OECD 2021 – Austria [79] | The State of Health in the EU’s Country Health Profiles provide a concise and policy-relevant overview of health and health systems in the EU/European Economic Area. The aim is to support policymakers and influencers with a means for mutual learning and voluntary exchange. | 1 | Policy and technical document | English | Austria | Rationalizing the HCWF distribution, Strengthening governance and Licensing and regulation | Text and opinion | Health and care workers not specified | Medium |
| OECD 2021 – Sweden [81] | The State of Health in the EU’s Country Health Profiles provide a concise and policy-relevant overview of health and health systems in the EU/European Economic Area. The aim is to support policymakers and influencers with a means for mutual learning and voluntary exchange. | 1 | Policy and technical document | English | Sweden | Optimizing roles, Improving health worker availability, and Strengthening governance | Text and opinion | Health and care workers not specified | High |
| OECD 2021 - North Macedonia [75] | The State of Health in the EU’s Country Health Profiles provide a concise and policy-relevant overview of health and health systems in the EU/European Economic Area. The aim is to support policymakers and influencers with a means for mutual learning and voluntary exchange. | 1 | Policy and technical document | English | North Macedonia | Building Competences through education and training, Improving health worker availability | Text and opinion | Health and care workers not specified | Medium |
| OECD 2020 - Empowering the health workforce [74] | This report consists of three parts, and outlines how digital technologies can help to address existing and emerging health policy challenges as well as how far the EU and OECD countries are in seizing these opportunities; discusses the health workforce related barriers and enablers to successful digital transformation; and describes a set of actions governments can take to activate the enablers and remove the barriers with the aim of empowering health workers to make the most of the digital revolution. | 1 | Policy and technical document | English | Denmark | Building Competences through education and training | Text and opinion | Health and care workers not specified | High |
| OECD 2021 – Germany [80] | The State of Health in the EU’s Country Health Profiles provide a concise and policy-relevant overview of health and health systems in the EU/European Economic Area. The aim is to support policymakers and influencers with a means for mutual learning and voluntary exchange. | 1 | Policy and technical document | English | Germany | Optimizing roles, Improving health worker availability, Rationalizing the HCWF distribution and Assessment, planning of HCWF needs | Text and opinion | Medical, nursing and health science students | Medium |
| Johnston et al. 2020 [99] | To explore the roles dentists have undertaken within the community setting and reflect on dentists' transferable skills, training, and personal experiences during redeployment. | 1 | Policy and technical document | English | United Kingdom | Building Competences through education and training and Optimizing roles | Text and opinion | Dentists and community nurses | High |
| Zhang et al 2021[57] | Describes the importance of organizational leadership, emergency psychological crisis interventions in pandemics, and psychological intervention measures for medical staff. | 1 | Policy and technical document | English | China | Decent working conditions | Text and opinion | Health and care workers not specified | Medium |
| OECD 2021 – Croatia [56] | The State of Health in the EU’s Country Health Profiles provide a concise and policy-relevant overview of health and health systems in the EU/European Economic Area. The aim is to support policymakers and influencers with a means for mutual learning and voluntary exchange. | 1 | Policy and technical document | English | Croatia | Decent working conditions | Text and opinion | Health and care workers not specified | Medium |
| OECD 2021 – Spain [55] | The State of Health in the EU’s Country Health Profiles provide a concise and policy-relevant overview of health and health systems in the EU/European Economic Area. The aim is to support policymakers and influencers with a means for mutual learning and voluntary exchange. | 1 | Policy and technical document | English | Spain | Decent working conditions, Improving health worker availability, Rationalizing the HCWF distribution and Strengthening governance | Text and opinion | Nurses, physicians and health and care workers not specified | Medium |
| OECD 2021 – Czechia [51] | The State of Health in the EU’s Country Health Profiles provide a concise and policy-relevant overview of health and health systems in the EU/European Economic Area. The aim is to support policymakers and influencers with a means for mutual learning and voluntary exchange. | 1 | Policy and technical document | English | Czechia | Improving health worker availability, and Rationalizing the HCWF distribution | Text and opinion | Nurses, physicians and health and care workers not specified | Medium |
| OECD 2021 – Iceland [50] | The State of Health in the EU’s Country Health Profiles provide a concise and policy-relevant overview of health and health systems in the EU/European Economic Area. The aim is to support policymakers and influencers with a means for mutual learning and voluntary exchange. | 1 | Policy and technical document | English | Iceland | Decent working conditions and Assessment, planning of HCWF needs | Text and opinion | Nurses, physicians and frontline healthcare staff and auxiliary | High |
| OECD 2021 – Cyprus [49] | The State of Health in the EU’s Country Health Profiles provide a concise and policy-relevant overview of health and health systems in the EU/European Economic Area. The aim is to support policymakers and influencers with a means for mutual learning and voluntary exchange. | 1 | Policy and technical document | English | Republic of Cyprus | Decent working conditions and Improving health worker availability | Text and opinion | Nurses, physicians and health and care workers not specified | Medium |
| OECD 2020 [76] | This policy brief investigates how countries responded to immediate shortages of workers during the COVID-19 crisis. | 1 | Policy and technical document | English | United Kingdom, France, Australia, Italy | Building Competences through education and training, Improving health worker availability, and Licensing and regulation | Text and opinion | Health and care workers not specified | High |
| Williams et al 2020 [73] | Explores the strategies that 44 countries in Europe plus Canada have taken to maintain and increase the availability of health workers. | 1 | Policy and technical document | English | Italy, Germany | Remuneration and incentives, Improving health worker availability, Strengthening governance and Licensing and regulation | Text and opinion | Nurses, physicians and health and care workers not specified | High |
| OECD 2021 – Poland [59] | The State of Health in the EU’s Country Health Profiles provide a concise and policy-relevant overview of health and health systems in the EU/European Economic Area. The aim is to support policymakers and influencers with a means for mutual learning and voluntary exchange. | 1 | Policy and technical document | English | Poland | Decent working conditions, Remuneration and incentives, Optimizing roles, Improving health worker availability, Rationalizing the HCWF distribution and Strengthening governance | Text and opinion | Physicians, nurses, paramedics and other medical | Medium |
| OECD 2021 – Luxembourg [43] | The State of Health in the EU’s Country Health Profiles provide a concise and policy-relevant overview of health and health systems in the EU/European Economic Area. The aim is to support policymakers and influencers with a means for mutual learning and voluntary exchange. | 1 | Policy and technical document | English | Luxembourg | Decent working conditions, Remuneration and incentives, Optimizing roles, Rationalizing the HCWF distribution and Assessment, planning of HCWF needs | Text and opinion | Health and care workers not specified | Medium |
| Ricci et al 2020 [72] | Highlights the main anti-COVID-19 government measures to support doctors and healthcare professionals, and it analyzes how to respond to the many requests complaining about neglectful healthcare professionals during the spread of the infection. | 1 | Policy and technical document | English | Italy | Remuneration and incentives, and Licensing and regulation | Text and opinion | Nurses, physicians and health and care workers not specified | Medium |
| Williams et al 2022 [77] | To summarize how digital health tools have been used to support the COVID-19 response across Europe. | 1 | Policy and technical document | English | Belgium, Denmark, Estonia, Finland, France, Germany, Ireland, Italy, Netherlands, Poland and Romania. | Building Competences through education and training, and Rationalizing the HCWF distribution | Text and opinion | Health and care workers not specified | High |
| World Health Organization 2021 [98] | The focus of this report is to share reflections, experiences and resources discussed during a three-part webinar series held by the WHO Regional Office for Europe towards the end of 2020. | 1 | Policy and technical document | English | Scotland | Decent working conditions | Text and opinion | Health and care workers not specified | Medium |
| Muhamad Amir et al 2021 [47] | Provide short-term relief and strategize on mobilizing ancillary medical and health personnel along with volunteers from the Ministry of Health of Malaysia, government and non-governmental bodies, private sectors, and individuals as part of the public health response to areas with the highest burden of COVID-19. | 1 | Policy and technical document | English | Malaysia | Decent working conditions, Improving HCWF and Strengthening governance | Text and opinion | Health and care workers not specified | High |
| Wong et al 2022 [46] | Viewpoint to reflect on the understanding and application of digital public health during the COVID-19 pandemic and in a wider context, as well as discuss the opportunities, challenges, and implications of the increasing utilized action of public health in Europe. | 1 | Policy and technical document | English | United Kingdom | Decent working conditions | Text and opinion | Health and care workers not specified | High |
| Rocard et al. 2021 [34] | The analysis presented in this report describes the effects of COVID-19 on LTC in OECD countries, takes stock of the wide range of policy responses that countries have implemented, assesses emergency preparedness in the sector, finally looks at how policy responses have affected continuity of care and the well-being of residents. | 1 | Policy and technical document | English | Austria, Japan, France and Canada | Decent working conditions, Remuneration and incentives, Optimizing roles, Rationalizing the HCWF distribution and Licensing and regulation | Text and opinion | Health and care workers not specified | Medium |
| Nittayasoot et al. 2021 [33] | Describe the nature of the health workforce and function that facilitated the capacity to respond to this pandemic, the public health policies and social interventions that allowed the virus to be successfully contained. | 1 | Policy and technical document | English | Thailand | Decent working conditions, and Remuneration and incentives | Text and opinion | Nurses, physicians and health and care workers not specified | High |
| Sagan et al 2021 [71] | Review of the evidence analyzed through the lens of the health system functions and examines how actions have contributed to strengthening health system responses. | 1 | Policy and technical document | English | Germany, United States, Australia, Canada, United Kingdom | Remuneration and incentives and Optimizing roles | Text and opinion | Physicians (GPS) and health and care workers not specified | High |
| OECD 2021 – Belgium [69] | The State of Health in the EU’s Country Health Profiles provide a concise and policy-relevant overview of health and health systems in the EU/European Economic Area. The aim is to support policymakers and influencers with a means for mutual learning and voluntary exchange. | 1 | Policy and technical document | English | Belgium | Remuneration and incentives, Optimizing roles, Improving health worker availability, Rationalizing the HCWF distribution, and Strengthening governance | Text and opinion | Dentist, nurses, physicians, pharmacist, physiotherapist and health and care workers not specified | Medium |
| Buchan et al. 2021 [60] | Consider some of the effective governance tools that have been utilized to mobilize, redeploy and repurpose the health workforce during the COVID-19 pandemic. | 1 | Policy and technical document | English | Kyrgyzstan, Ireland, and Scotland | Decent working conditions and Improving HCWF information systems | Text and opinion | Health and care workers not specified | High |
| Betliy 2021 [68] | This report reviews Ukraine’s social policy response to the Covid-19 pandemic between March and September 2020. | 1 | Policy and technical document | English | Ukraine | Remuneration and incentives | Text and opinion | Health and care workers not specified | Medium |
| Waitzberg et al 2020 [67] | To support policymakers across countries in tailoring policies to tackle health provider´s loss of income during the COVID-19 pandemic. | 1 | Policy and technical document | English | England, Netherlands and Estonia | Remuneration and incentives and Rationalizing the HCWF distribution | Text and opinion | Physicians and health and care workers not specified | Low |
| OECD 2021 – Slovakia [66] | The State of Health in the EU’s Country Health Profiles provide a concise and policy-relevant overview of health and health systems in the EU/European Economic Area. The aim is to support policymakers and influencers with a means for mutual learning and voluntary exchange. | 1 | Policy and technical document | English | Slovakia | Remuneration and incentives, Rationalizing the HCWF distribution, Strengthening governance, and Licensing and regulation | Text and opinion | Nurses, physicians and health and care workers not specified | Medium |
| OECD 2021 – Lithuania [65] | The State of Health in the EU’s Country Health Profiles provide a concise and policy-relevant overview of health and health systems in the EU/European Economic Area. The aim is to support policymakers and influencers with a means for mutual learning and voluntary exchange. | 1 | Policy and technical document | English | Lithuania | Remuneration and incentives, Improving health worker availability, and Licensing and regulation | Text and opinion | Health and care workers not specified | High |
| OECD 2021 – Slovenia [64] | The State of Health in the EU’s Country Health Profiles provide a concise and policy-relevant overview of health and health systems in the EU/European Economic Area. The aim is to support policymakers and influencers with a means for mutual learning and voluntary exchange. | 1 | Policy and technical document | English | Slovenia | Remuneration and incentives and Improving health worker availability | Text and opinion | Nurses, physicians and health and care workers not specified | High |
| OECD 2021 – Romania [54] | The State of Health in the EU’s Country Health Profiles provide a concise and policy-relevant overview of health and health systems in the EU/European Economic Area. The aim is to support policymakers and influencers with a means for mutual learning and voluntary exchange. | 1 | Policy and technical document | English | Romania | Decent working conditions, Remuneration and incentives, Optimizing roles, Improving health worker availability, Rationalizing the HCWF distribution and Strengthening governance | Text and opinion | Nurses, physicians and health and care workers not specified | Medium |
| OECD 2021 – Latvia [53] | The State of Health in the EU’s Country Health Profiles provide a concise and policy-relevant overview of health and health systems in the EU/European Economic Area. The aim is to support policymakers and influencers with a means for mutual learning and voluntary exchange. | 1 | Policy and technical document | English | Latvia | Decent working conditions, Remuneration and incentives and Improving health worker availability | Text and opinion | Nurses, physicians, pharmacist and health and care workers not specified | High |
| Williams et al. 2020 [58] | Explore the range of mental health, financial and other practical support measures that 36 countries in Europe and Canada have put in place. | 1 | Policy and technical document | English | 36 countries in Europe and Canada - data extracted from - Malta, Poland, Lithuania, France, Italy, Bulgaria, Kyrgyzstan and France, Turkey and Hungary, Italy, Poland | Decent working conditions and Remuneration and incentives | Text and opinion | Physicians, nurses, paramedics and other medical | Medium |
| OECD 2021 – Estonia [44] | The State of Health in the EU’s Country Health Profiles provide a concise and policy-relevant overview of health and health systems in the EU/European Economic Area. The aim is to support policymakers and influencers with a means for mutual learning and voluntary exchange. | 1 | Policy and technical document | English | Estonia | Decent working conditions, Remuneration and incentives, Improving health worker availability, Rationalizing the HCWF distribution and Assessment, planning of HCWF needs | Text and opinion | Nurses, physicians and other | Medium |
| OECD 2021 [70] | A policy brief that describes how the COVID-19 pandemic is impacting the lives of people with chronic conditions and how primary health care systems play an essential role in response to the pandemic. | 1 | Policy and technical document | English | 30 countries - data extracted from Scotland, United States, France, Germany, Belgium, Australia, Greece, Netherlands, United Kingdom, Canada | Remuneration and incentives, Optimizing roles and Rationalizing the HCWF distribution | Text and opinion | Physicians, community phamacists and psychotherapists | High |
| OECD 2021 – Hungary [62] | The State of Health in the EU’s Country Health Profiles provide a concise and policy-relevant overview of health and health systems in the EU/European Economic Area. The aim is to support policymakers and influencers with a means for mutual learning and voluntary exchange. | 1 | Policy and technical document | English | Hungary | Remuneration and incentives, Improving health worker availability and Rationalizing the HCWF distribution | Text and opinion | Dentist, nurses, physicians, pharmacist and health and care workers not specified | Medium |
| OECD 2021 – Greece [52] | The State of Health in the EU’s Country Health Profiles provide a concise and policy-relevant overview of health and health systems in the EU/European Economic Area. The aim is to support policymakers and influencers with a means for mutual learning and voluntary exchange. | 1 | Policy and technical document | English | Greece | Decent working conditions, Remuneration and incentives, Improving health worker availability, Rationalizing the HCWF distribution and Strengthening governance | Text and opinion | Health and care workers not specified | Medium |
| Williams et al. 2022 [94] | Provide an overview of the strategies that have been adopted to support health workers, both in clinical settings and outside. | 1 | Policy and technical document | English | Poland and France | Supportive work environment and manageable workload | Text and opinion | Health and care workers not specified | High |
| OECD 2021 – Bulgaria [101] | The State of Health in the EU’s Country Health Profiles provide a concise and policy-relevant overview of health and health systems in the EU/European Economic Area. The aim is to support policymakers and influencers with a means for mutual learning and voluntary exchange. | 1 | Policy and technical document | English | Bulgaria | Remuneration and incentives | Text and opinion | Physicians | Medium |
| Ares Blanco 2021 [97] | To describe the management of human resource and the vaccination strategies in primary care in twelve European countries in relation to the COVID-19 pandemic. | 1 | Article | Spanish | Spain | Licensing and regulation | Qualitative | Physicians | Low |
| OECD 2020 [96] | Presents the contribution of migrant doctors and nurses to OECD health systems and how OECD countries have adapted the recognition of foreign credentials to mobilize additional doctors and nurses with foreign degrees in response to COVID‑19. | 1 | Policy and technical document | English | Canada | Licensing and regulation | Text and opinion | Physicians | High |
| WHO 2022 [42] | Summarizes key findings and looks at the impact of COVID-19 on the health workforce, occupational safety and working conditions of the HWF and on the strategies and mechanisms used by these countries to increase, maintain and protect HRH, in terms of their availability, training, protection, welfare, remuneration and financing. | 1 | Policy and technical document | English | Bolivia, Chile, Colombia, Ecuador and Peru | Decent working conditions, Remuneration and incentives, Optimizing roles, Improving health worker availability, Rationalizing the HCWF distribution, Strengthening governance and Licensing and regulation | Qualitative | Health and care workers not specified | Medium |
| Rees et at 2021 [63] | To recount the progress that the Peruvian government has been making to improve its workforce capacity and planning and begin to answer the question of how Peru's health workforce policy and planning is addressing health system pressures arising from the pandemic? | 1 & 2 | Article | English | Peru | Remuneration and incentives and Improving health worker availability | Qualitative | Professional assistant, technical assistant, administrative, physicians (including resident doctors), Nurse and Midwife, foreign medical personnel | Medium |
| Hernandez-Perez 2021 [32] | To evaluate the effectiveness of educational intervention on COVID-19 knowledge and biosafety in care nurses exposed to SARSCoV-2. | 1 & 2 | Article | Spanish | Cuba | Building Competences through education and training | Quasi-experimental | Nurses | High |
| Hou et al 2020 [30] | To identify the effectiveness of a personnel protection strategy in protection of healthcare workers from SARS-CoV-2 infection. | 1 & 2 | Article | English | China | Decent working conditions | Cohort Studies | Nurses, physicians and other | Medium |
| Vogazianos et al 2021 [45] | Present the first National multimodal quality and safety improvement strategy plan for the long-term care facilities in the Republic of Cyprus. | 1 & 2 | Article | English | Republic of Cyprus | Decent working conditions | Quasi-experimental | Health and care workers not specified | Medium |
| Otu et al 2021[28] | Development of InStrat COVID-19 tutorial app, to deliver accurate and regularly updated information about COVID-19 to frontline health workers and epidemic response officers across 25 states of Nigeria. | 1 & 2 | Article | English | Nigeria | Building Competences through education and training | Cross-Sectional | Physicians, nurses, midwives, laboratory technicians, pharmacists, community health officers (CHO) and Community Health Extension Workers (CHEW) | Medium |
| Brito-Brito et al 2021 [35] | To analyze the impact of an online training intervention on primary healthcare professionals in Tenerife (Canary Islands, Spain), evaluating the perceived knowledge about prevention and control of SARS-CoV-2 infection using the NOC outcome “Knowledge: Infection management". | 1 & 2 | Article | English | Tenerife Island (Spain) | Building Competences through education and training | Quasi-experimental | Nurses, physicians, nursing assistants, hospital porters, dentists, oral hygiene technicians, pharmacists, and physiotherapists non-health professionals (administrative assistant social workers) | Medium |
| Abbas et al. 2021 [31] | To explore working dynamics and experience of junior and middle grade doctors during current pandemic. | 1 & 2 | Article | English | United Kingdom | Rationalizing the HCWF distribution | Cross-Sectional | Physicians | Medium |
| O´Neil et al 2021 [29] | To characterize the activity of teleconsultation/medical consultation without the presence of the patient, during the first phase of the COVID-19 pandemic, to assess the perception of doctors working in the National Health Service (NHS) regarding teleconsultation, and to investigate the feasibility of teleconsultation as a complement or regular alternative to face-to-face consultation, after the pandemic. | 1 & 2 | Article | English | Portugal | Rationalizing the HCWF distribution | Qualitative | Physicians | High |
