# Supplementary material for: Implementation of policy and management interventions to improve health and care workforce capacity to address the COVID-19 pandemic response: a systematic review
Source: Hum Resour Health. 2023 Oct 10;21:80. doi: 10.1186/s12960-023-00856-y (PMC10563305; doi:10.1186/s12960-023-00856-y)
Supplement: Supplementary file 5 — Additional file 5. Tables of excluded documents. [file 12960_2023_856_MOESM5_ESM.docx]

**Additional file 5 - Tables of excluded documents (from databases and websites and reasons for exclusion)**

***Table of excluded documents from databases and reasons for exclusion (n=171)***

| Authors | Title | Reason |
| --- | --- | --- |
| Agrawal et.al | Resource husbandry in challenging times | Type of document |
| Ahmad et.al | Occupational Health and Safety Measures in Healthcare Settings during COVID-19: Strategies for Protecting Staff, Patients and Visitors | Intervention |
| Alaradi et.al | The Mental Health Impact of COVID-19 Pandemic on Health Care Workers and Coping Strategies: A Systematic Literature Review | Intervention |
| Alfonso et.al | Early Interventions and Impact of COVID-19 in Spain | Population |
| Ali et.al | The Strategic Role of Healthcare Professionals in the Battle Against COVID-19: Do We Really Need Help from Community Pharmacists? | Type of document |
| Aminah et.al | The barriers of policy implementation of handling Covid-19 pandemic in Indonesia | Intervention |
| Anderson et.al | Securing a sustainable and fit-for-purpose UK health and care workforce | Intervention |
| Aron et.al | Strategies for responding to the COVID-19 pandemic in a rural health system in New York state | Intervention |
| Aydogdu | Pandemic caused by the new coronavirus: health system and coping measures in Turkey | Population |
| Babaei et.al | Interventions for adapting health care providers to new situations in the workplace during the COVID-19 pandemic: A scoping review for developing a policy brief | Context |
| Barbosa et.al | Strategies to reorganize hospital care in Paraná in confronting Covid-19 | Population |
| Barlas et.al | Turkey's response to COVID-19 pandemic: strategy and key actions | Intervention |
| Bertuzzi et.al | Psychological Support Interventions for Healthcare Providers and Informal Caregivers during the COVID-19 Pandemic: A Systematic Review of the Literature | Intervention |
| Bhaumik et.al | Community health workers for pandemic response: a rapid evidence synthesis | Intervention |
| Cairns et.al | Interventions for the well-being of healthcare workers during a pandemic or other crisis: scoping review | Context |
| Claeys et.al | Evaluation of the medication policy during and after a stay in Covid transitional care centers in Flanders | Intervention |
| Clark et.al | A systematic review of de-escalation strategies for redeployed staff and repurposed facilities in COVID-19 intensive care units (ICUs) during the pandemic | Context |
| Coates et.al | Health workforce strategies in response to major health events: a rapid scoping review with lessons learned for the response to the COVID-19 pandemic | Context |
| Cruz, et.al | The strategies for early detection and blocking the transmission of covid-19 in the workers of the Department of Health of the State of Bahia | Intervention |
| Dahn et.al | Liberia's First Health Workforce Program Strategy: Reflections and Lessons Learned | Context |
| Dhandapani et.al | Challenges posed by COVID-19 and neurosurgical nursing strategies in developing countries | Type of document |
| Dhuria et.al | Is India ready to address covid-19 like pandemics: A perspective from existing public health acts | Type of document |
| Diver et.al | The value of healthcare worker support strategies to enhance wellbeing and optimise patient care | Intervention |
| Duan et.al | Personal Protective Equipment in COVID-19: Impacts on Health Performance, Work-Related Injuries, and Measures for Prevention | Intervention |
| Duim, et.al | Caring for the Workforce of a Health System During the COVID-19 Epidemic in Brazil: Strategies of Surveillance and Expansion of Access to Care | Intervention |
| Dullius et.al | Coping strategies in health professionals facing Covid-19: s systematic review | Intervention |
| Eisele et.al | Strategies in Primary Care to Face the SARS-CoV-2 / COVID-19 Pandemic: An Online Survey | Intervention |
| El Bizri et.al | The role of community pharmacists in increasing access and use of self-care interventions for sexual and reproductive health in the Eastern Mediterranean Region: examples from Egypt, Jordan, Lebanon and Somalia | Context |
| Elbeddini et.al | Pharmacist intervention amid the coronavirus disease 2019 (COVID-19) pandemic: From direct patient care to telemedicine | Type of document |
| Elgibaly et.al | Knowledge, perception, and confidence of healthcare workers about COVID-19 preventive measures during the first wave of the pandemic: A cross-sectional study from Egypt | Intervention |
| Elizarrarás-Rivas et.al | Measures for the protection of health personnel in the coronavirus pandemic (COVID-19) | Intervention |
| Elsayed et.al | Non-consultant hospital doctors views' of covid-19 measures in Irish maternity units | Intervention |
| Emanuel et.al | Mandating COVID-19 Vaccination for Health Care Workers | Type of document |
| Enabulele et.al | The risk perception of COVID-19 and practice of precautionary measures amongst healthcare workers in the National Health Insurance Scheme Clinic of a tertiary hospital in Nigeria | Intervention |
| Epler et.al | ICU telehealth use and concern for workforce shortages among rural hospitals in Michigan at the onset of the COVID-19 pandemic | Type of document |
| Eum et.al | The Role of Information and Communications Technology Policies and Infrastructure in Curbing the Spread of the Novel Coronavirus: Cross-country Comparative Study | Population |
| Evans et.al | The impact of testing and infection prevention and control strategies on within-hospital transmission dynamics of COVID-19 in English hospitals | Intervention |
| Everhart et.al | Categorization of full-time tele-critical care pharmacist interventions based on shift | Type of document |
| Farooqi et.al | An overview of SARS-COV-2 epidemiology, mutant variants, vaccines, and management strategies | Population |
| Feroz et.al | Equipping community health workers with digital tools for pandemic response in LMICs | Type of document |
| Ferrara, et.al | The cost of caring during recent epidemics: a rapid review of risk factors, psychological manifestations, and strategies for its treatment | Context |
| Fong et.al | Evaluating the longitudinal effectiveness of preventive measures against COVID-19 and seroprevalence of IgG antibodies to SARS-CoV-2 in cancer outpatients and healthcare workers | Intervention |
| Fortuna et.al | Strategies to increase peer support specialists' capacity to use digital technology in the era of COVID-19: Pre-post study | Intervention |
| Foster, S | Leadership in the time of crisis | Type of document |
| Foster, S | Considering care in context | Type of document |
| Fredericks et.al | Identifying evidence informed psychological interventions during the COVID-19 pandemic: Rapid review of the literature | Intervention |
| Gadsby et.al | Qualitative analysis of the impact of the SARS-CoV-2 pandemic response on paediatric health services in North of Scotland and North of England | Intervention |
| Galvin, M C | Shaping the future of healthcare: building back better | Type of document |
| Garattini et.al | Improving primary care in Europe beyond COVID-19: from telemedicine to organizational reforms | Type of document |
| Giancotti et.al | The role of European health system characteristics in affecting Covid 19 lethality during the early days of the pandemic | Population |
| Gomes, et.al | Protective, administrative, and environmental strategies for workers' health during the pandemic | Context |
| Gonçalves, et.al | Coping strategies and health promotion through teaching-service integration in the context of the COVID-19 pandemic | Intervention |
| Gong et.al | Integrated Healthcare Systems Response Strategies Based on the Luohu Model During the COVID-19 Epidemic in Shenzhen, China | Intervention |
| Gross et.al | COVID-19 and healthcare workers: a rapid systematic review into risks and preventive measures | Context |
| Gyanwali et.all | Assessment of Preparedness of Government of Nepal in COVID Designated Hospitals and Clinics for Pandemic Response | Intervention |
| Hasan et.al | The implementation of mass-vaccination against SARS-CoV-2: A systematic review of existing strategies and guidelines | Population |
| Hasan, et.al | Social distancing and the use of PPE by community pharmacy personnel: Does evidence support these measures? | Intervention |
| Health Ministry Peru | Dictan medidas temporales para asegurar el suministro de productos necesarios para la salud durante la emergencia sanitaria declarada como consecuencia del COVID-19 | Type of document |
| Health Ministry Peru | Decreto Legislativo que establece medidas temporales excepcionales en materia de gestión de recursos humanos en el sector público ante la emergencia sanitaria ocasionada por el COVID-19 | Population |
| Hussain et.al | Protecting healthcare workers from COVID-19: learning from variation in practice and policy identified through a global cross-sectional survey | Intervention |
| Instituto Nacional de Salud | Protective measures in health personnel for the prevention of COVID-19 vs2 | Type of document |
| Izzetti et.al | COVID-19 Transmission in Dental Practice: Brief Review of Preventive Measures in Italy | Intervention |
| Jafree et.al | WhatsApp-Delivered Intervention for Continued Learning for Nurses in Pakistan During the COVID-19 Pandemic: Results of a Randomized-Controlled Trial | Intervention |
| Jesuthasan et.al | We weren't checked in on, nobody spoke to us': an exploratory qualitative analysis of two focus groups on the concerns of ethnic minority NHS staff during COVID-19 | Intervention |
| Juvet et.al | Adapting to the unexpected: Problematic work situations and resilience strategies in healthcare institutions during the COVID-19 pandemic's first wave | Intervention |
| Kamberi et.al | Impact of COVID-19 pandemic on mental health, risk perception and coping strategies among health care workers in Albania - evidence that needs attention | Intervention |
| Kapitsinis, N | The underlying factors of excess mortality in 2020: a cross-country analysis of pre-pandemic healthcare conditions and strategies to cope with Covid-19 | Population |
| Karimi et. Al | Interventions to Improve the Willingness to Work Among Health care Professionals in Times of Disaster: A Scoping Review | Intervention |
| Kaur et.al | Emerging issues and preventive measures for health care workers in the diagnostic field in coronavirus disease 2019 pandemic | Intervention |
| Kisting-Cairncross, S | Review of Respiratory Protections for Health Workers: Environmental, Administrative and Personal Protective Measures and the Controversies Surrounding These | Type of document |
| Kleinpell et.al | Coronavirus Disease 2019 Pandemic Measures: Reports from a National Survey of 9,120 ICU Clinicians | Intervention |
| Klompas et.al | The Case for Mandating COVID-19 Vaccines for Health Care Workers | Type of document |
| Klumpp et.al | COVID-19 health policy evaluation: integrating health and economic perspectives with a data envelopment analysis approach | Population |
| Kokou et.al | The "Healthwise" Approach In Togo, From Pilot Centers To The National Strategy For The Promotion Of Safety And Health At Work For Health Workers (2016-2021) | Type of document |
| Kraus et.al | Practical Solutions for Healthcare Worker Protection During the COVID-19 Pandemic Response in the Ambulatory, Emergency, and Inpatient Settings | Intervention |
| Ku et.al | Provision of a consistent national approach to radiation therapy workforce protection measures in Australia during the COVID-19 pandemic | Type of document |
| Kua et.al | The coping strategies of community pharmacists and pharmaceutical services provided during COVID-19 in Malaysia | Intervention |
| Laestadius et.al | UN-Wide Health Survey: Data Driven Strategies for Post-Pandemic Recovery and Resilience | Type of document |
| Lotta et.al | The vulnerabilities of the Brazilian health workforce during health emergencies: Analysing personal feelings, access to resources and work dynamics during the COVID-19 pandemic | Intervention |
| Maleki et.al | Insecure Employment Contracts during the COVID-19 Pandemic and the Need for Participation in Policy Making | Type of document |
| Martín-Aragón-Gelabert et.al | Post-COVID-19 psychosocial intervention in healthcare professionals (Intervención psicosocial postCOVID-19 en personal sanitario) | Intervention |
| Martindale et.al | Perspectives on COVID-19 testing policies and practices: a qualitative study with scientific advisors and NHS health care workers in England | Intervention |
| Martínez et.al | Estimating patient empowerment and nurses' use of digital strategies: eSurvey study | Population |
| Marya et.al | The Exponential Rise of Teledentistry and Patient-Oriented Protective Measures in Southeast Asian Dental Clinics: Concerns, Benefits, and Challenges | Population |
| Mash et.al | Re-organising primary health care to respond to the Coronavirus epidemic in Cape Town, South Africa | Population |
| Matoori et.al | Preparing for future waves and pandemics: a global hospital survey on infection control measures and infection rates in COVID-19 | Intervention |
| Mehta et.al | Strategies to prevent burnout in the cardiovascular health-care workforce | Type of document |
| Méndez-Rí­os , J | Minimum Biosafety Measures in healthcare facilities during SARS­CoV­2 Pandemic | Intervention |
| Mercer et.al | COVID-19 pandemic vaccination preparedness strategies for independent community pharmacies | Intervention |
| Mezue et.al | Sub-Saharan Africa Tackles COVID-19: Challenges and Opportunities | Type of document |
| Miele et.al | Telemedicine in Parkinson's Disease: How to Ensure Patient Needs and Continuity of Care at the Time of COVID-19 Pandemic | Population |
| Minnie, D | Global Cry for Strategies to Support the Mental Health and Well Being of Health Care Workers | Type of document |
| Mishra et.al | Resolution of Resilience: Empirical Findings on the Challenges Faced and the Mitigation Strategies Adopted by Community Health Workers to Provide Maternal and Child Health (MCH) Services during the COVID-19 Pandemic in the Context of Odisha, India | Intervention |
| Moey et.al | What are the measures taken to prevent COVID-19 infection among healthcare workers? A retrospective study in a cluster of primary care clinics in Singapore | Intervention |
| Mohammed et.al | Stress coping strategies among critical care medicine physicians during covid-19 pandemic in Egypt: A qualitative study | Intervention |
| Mohanty et.al | Coronavirus Disease-19 Testing Strategies for Patients and Health Care Workers to Improve Workplace Safety | Intervention |
| Mohapatra et.al | Effective intervention & workplace wellness of healthcare providers: Perspective from Odisha | Type of document |
| Moni et.al | Waiting for godot: A cross sectional survey based analysis of the hydroxychloroquine prophylaxis strategy against covid-19 in India | Intervention |
| Monteblanco, A D | The COVID-19 pandemic: A focusing event to promote community midwifery policies in the United States | Intervention |
| Montgomery et.al | Development and Implementation of a Pediatric Telesimulation Intervention for Nurses in Community Emergency Departments | Intervention |
| Moreira et.al | Mental health interventions implemented in the COVID-19 pandemic: what is the evidence? | Context |
| Morganstein et.al | Enhancing Psychological Sustainment & Promoting Resilience in Healthcare Workers During COVID-19 & Beyond: Adapting Crisis Interventions from High-Risk Occupations | Intervention |
| Moseley et.al | Mitigating the Transmission of COVID-19 with the Appropriate Usage of Personal Protective Protocols and Equipment in Breast Imaging and Intervention | Intervention |
| Mukhamedyarova et.al | Measures to strengthen the role of primary care nurses during the COVID-19 pandemic: A concept analysis | Intervention |
| Mukherjee et.al | Impact of the COVID-19 pandemic on the human resources for health in India and key policy areas to build a resilient health workforce | Type of document |
| Mukherjee et.al | The urologist, personal protective equipment (PPE) and covid-19: A review of current challenges around the use of PPE as well as measures to reduce the risk of nosocomial covid-19 transmission | Intervention |
| Muller et.al | The mental health impact of the covid-19 pandemic on healthcare workers, and interventions to help them: A rapid systematic review | Context |
| Nascimento et.al | Strategies for worker's mental health maintenance in Covid-19 times: An Integrative Review | Context |
| Nath et.al | Capacity development and safety measures for health care workers exposed to COVID-19 in Bangladesh | Intervention |
| Neumann et.al | Workplace Health Promotion and COVID-19 Support Measures in Outpatient Care Services in Germany: A Quantitative Study | Intervention |
| Neupane et.al | COVID 19 and Nepal: Identification of Critical Public Health Measures | Type of document |
| Nigris et.al | The training strategy in POCT governance | Type of document |
| Nishiyama et.al | Labour, ethical and political dimensions of nursing staff sizing in the face of COVID-19 | Type of document |
| Novak et.al | Pharmacists' role, work practices, and safety measures against COVID-19: A comparative study | Intervention |
| Nyabi et.al | Diagnostic Value of IgM and IgG Detection in COVID-19 Diagnosis by the Mobile Laboratory B-Life: A Massive Testing Strategy in the Piedmont Region | Intervention |
| Ou et.al | Community Pharmacists in Taiwan at the Frontline Against the Novel Coronavirus Pandemic: Gatekeepers for the Rationing of Personal Protective Equipment | Type of document |
| PAHO | La adaptación del primer nivel de atención en el contexto de la pandemia de COVID-19: intervenciones, modalidades y ámbitos, 23 de abril del 2020 | Type of document |
| Pathak et.al | Perception and Challenges of Preventive Measures of COVID-19 Among Nepalese Frontline Health Professionals: An Unexplored Realism | Intervention |
| Paudyal, et.al | Pharmacists' involvement in COVID-19 vaccination across Europe: a situational analysis of current practice and policy | Type of document |
| Peine et.al | Telemedicine in Germany During the COVID-19 Pandemic: Multi-Professional National Survey | Intervention |
| Pereira et.al | Heterogeneidades das pol\~AÂ­ticas estaduais de distanciamento social diante da COVID-19: aspectos pol\~AÂ­ticos e t\~A\textcopyrightcnico-administrativos | Population |
| Pereira, et al | COVID-19 and strategies to reduce anxiety in nursing: scoping review and meta-analysis | Intervention |
| Persad et.al | Elimination, substitution, engineering, and administrative interventions to reduce the risk of SARS-CoV-2 infection in healthcare workers | Type of document |
| Petersen et.al | COVID-19-We urgently need to start developing an exit strategy | Population |
| Pilbeam et.al | How do Healthcare Workers 'Do' Guidelines? Exploring How Policy Decisions Impacted UK Healthcare Workers During the First Phase of the COVID-19 Pandemic | Intervention |
| Pilbeam et.al | How do Healthcare Workers 'Do' Guidelines? Exploring How Policy Decisions Impacted UK Healthcare Workers During the First Phase of the COVID-19 Pandemic | Intervention |
| Pollock et.al | Interventions to support the resilience and mental health of frontline health and social care professionals during and after a disease outbreak, epidemic or pandemic: a mixed methods systematic review | Intervention |
| Powell, H E | Dentists deployed: an insider's perspective of life on the NHS front line | Intervention |
| Price et.al | An educational intervention to increase advance care planning activities among emergency medicine providers during the covid-19 pandemic | Type of document |
| Priede et.al | Mental health interventions for healthcare workers during the first wave of COVID-19 pandemic in Spain | Intervention |
| Roberts et.al | The Impact of COVID-19 on Illinois Early Intervention Services | Population |
| Roberts et.al | Mental health and well-being impacts of COVID-19 on rural paramedics, police, community nurses and child protection workers | Intervention |
| Roche et.al | Learning lessons from the paediatric critical care response to the SARS-CoV-2 pandemic in England and Wales: a qualitative study | Intervention |
| Rowan et.al | Challenges and solutions for addressing critical shortage of supply chain for personal and protective equipment (PPE) arising from Coronavirus disease (COVID19) pandemic - Case study from the Republic of Ireland | Intervention |
| Rykers et.al | Victoria (Australia) radiotherapy response to working through the first and second wave of COVID-19: Strategies and staffing | Intervention |
| Sahoo et.al | Urban to rural COVID-19 progression in India: The role of massive migration and the challenge to India's traditional labour force policies | Population |
| SaideI et.al | Mental health interventions for health professionals in the context of the Coronavirus pandemic | Intervention |
| Saran et.al | Identifying and Implementing Strategies to Reduce the Risk of Self-Contamination of Health Care Workers Caused by Doffing of Personal Protective Equipment During the COVID-19 Pandemic | Intervention |
| Sarkar et.al | Public health preparedness and responses to the coronavirus disease 2019 (COVID-19) pandemic in South Asia: a situation and policy analysis | Population |
| Sato et.al | COVID-19 Mental Health Stressors of Health Care Providers in the Pandemic Acceptance and Commitment to Empowerment Response (PACER) Intervention: A Qualitative Study | Intervention |
| Sharp et.al | Policy and planning for large epidemics and pandemics - challenges and lessons learned from COVID-19 | Population |
| Shrestha et.al | Health System Preparedness for COVID-19 and Its Impacts on Frontline Health-Care Workers in Nepal: A Qualitative Study Among Frontline Health-Care Workers and Policy-Makers | Intervention |
| Silva et.al | Biosafety measures to prevent COVID-19 in healthcare professionals: an integrative review | Intervention |
| Smith et.al | Adherence to protective measures among healthcare workers in the UK: a cross-sectional study | Intervention |
| Spetz, J | Changes in registered nurse employment and education capacity during the COVID-19 pandemic and the risk of future shortages | Type of document |
| Stephen et.al | Scrutiny of COVID-19 response strategies among severely affected European nations | Population |
| Subramaniam et.al | Development and validation of a tool to appraise guidelines on SARS-CoV-2 infection control strategies in healthcare workers | Intervention |
| Temkin et.al | Effect of a national policy of universal masking and uniform criteria for severe acute respiratory coronavirus virus 2 (SARS-CoV-2) exposure on hospital staff infection and quarantine | Intervention |
| Tian et.al | Risk factors and protective measures for healthcare worker infection during highly infectious viral respiratory epidemics: A systematic review and meta-analysis | Context |
| Torra-Bou et.al | Incidence, typology and measures of prevention and treatment of skin lesions associated with the use of personal protective equipment in health care professionals during the COVID-19 pandemic in Spain Collaborative study | Intervention |
| Treacy et.al | Repurposing psychological interventions for healthcare workers during COVID-19 | Type of document |
| Tsonis et.al | Psychological burden of covid-19 health crisis on health professionals and interventions to minimize the effect: what has history already taught us? | Intervention |
| van den Besselaar et.al | Implementation of a national testing policy in Dutch nursing homes during SARS-CoV-2 outbreaks | Population |
| van der Molen et.al | Following Dutch healthcare professionals' experiences during COVID-19: Tensions in everyday practices and policies amid shifting uncertainties | Intervention |
| van der Plaat et.al | Impact of COVID-19 pandemic on sickness absence for mental ill health in National Health Service staff | Intervention |
| Velana et.al | Individual-Level Interventions for Decreasing Job-Related Stress and Enhancing Coping Strategies Among Nurses: A Systematic Review | Intervention |
| Vicary et.al | "It's about how much we can do, and not how little we can get away with": Coronavirus-related legislative changes for social care in the United Kingdom | Intervention |
| Vimercati et.al | Prevention and protection measures of healthcare workers exposed to SARS-CoV-2 in a university hospital in Bari, Apulia, Southern Italy | Type of document |
| Warraich et.al | Medical leadership in the NHS during the COVID-19 pandemic | Type of document |
| Watkins et.al | Primary-care registered nurse telehealth policy implications | Context |
| Waya et.al | COVID-19 case management strategies: what are the options for Africa? | Type of document |
| Weston, M J | Strategic Planning for a Very Different Nursing Workforce | Context |
| Wise, S | Staffing policy in aged care must look beyond the numbers | Type of document |
| Wong et.al | Rebuilding to shape a better future: the role of young professionals in the public health workforce | Type of document |
| Wu et.al | COVID-19: Peer Support and Crisis Communication Strategies to Promote Institutional Resilience | Type of document |
| Xu et.al | Shortages of Staff in Nursing Homes During the COVID-19 Pandemic: What are the Driving Factors? | Intervention |
| Yetim et.al | Prioritization of the policies and practices applied in Turkey to fight against covid-19 through AHP technique | Intervention |
| Yoon et.al | A Systematic Narrative Review of Comprehensive Preparedness Strategies of Healthcare Resources for a Large Resurgence of COVID-19 Nationally, with Local or Regional Epidemics: Present Era and Beyond | Intervention |
| Yusuf et.al | Gaps in the implementation of COVID-19 mitigation measures could lead to development of new strains of antimicrobial resistant pathogens: Nigerian perspective | Type of document |
| Zaçe et.al | Interventions to address mental health issues in healthcare workers during infectious disease outbreaks: A systematic review | Context |
| Zhang et.al | Mechanism and prevention of facial pressure injuries: A novel emergent strategy supported by a multicenter controlled study in frontline healthcare professionals fighting COVID-19 | Intervention |

***Table of excluded documents from international organization websites and reasons for exclusion (n=41)***

| Authors | Title | Reasons for exclusion |
| --- | --- | --- |
| Bhaskar et.al | Telemedicine as the New Outpatient Clinic Gone Digital: Position Paper from the Pandemic Health System Resilience Program (REPROGRAM) International Consortium (Part 2) | Intervention |
| Busseli et.al | Mental health of Health Care Workers (HCWs): a review of organizational interventions put in place by local institutions to cope with new psychosocial challenges resulting from COVID-19 | Intervention |
| Chakraborty et.al | Violence against physicians and nurses: a systematic literature review | Context |
| Cormi et.al | Telepsychiatry to provide mental health support to healthcare professionals during the covid-19 crisis: A cross-sectional survey among 321 healthcare professionals in France | Intervention |
| Dubois et.al | COVID-19 and older people: Impact on their lives, support and care | Population |
| Dye et.al | Risk of COVID-19-related bullying, harassment and stigma among healthcare workers: An analytical cross-sectional global study | Intervention |
| Dzakula et.al | Croatia: Health System in transition | Intervention |
| Fahy et.al | European Support for improving health and care systems | Type of document |
| Fahy et.al | Use of digital tools in Europe: before, during and after COVID-19 | Population |
| Figueroa et.al | The Need for a Mental Health Technology Revolution in the COVID-19 Pandemic | Type of document |
| Fisk et.al | Telehealth in the Context of COVID-19: Changing Perspectives in Australia, the United Kingdom, and the United States | Population |
| Gerkens et. Al | Belgium: Health system in transition | Context |
| Gerson, Daniel and Mulligan, Donal | Public Servants and The Coronavirus (Covid-19) Pandemic: Emerging Responses and Initial Recommendations | Population |
| Gómez-Ibáñez et.al | Final-year nursing students called to work: Experiences of a rushed labour insertion during the COVID-19 pandemic | Intervention |
| Greenberg, N. and Weston, D. and Hall, C. and Caulfield, T. and Williamson, V. and Fong, K. | Mental health of staff working in intensive care during Covid-19 | Intervention |
| ICN | COVID-19and The International supply of Nurses | Intervention |
| ICN | International Council of Nurses: A Voice to Lead a Vision for Future Healthcare International Nurses Day 2021 Resources and Evidence | Intervention |
| ILO | Covid and the World of Work: Rapid assessment of the employment impacts and policy responses - North Macedonia | Population |
| ILO | Protecting the life and health of workers during the COVID-19 pandemic: Overview of national legislative and policy responses | Population |
| Merks et.al | The legal extension of the role of pharmacists in light of the COVID-19 global pandemic | Intervention |
| Oliveira, Tiago Cravo | Bringing health care to the patient: An overview of the use of telemedicine in OECD countries | Intervention |
| Organisation for Economic Co-operation and Development (OECD) | COVID-19 and key workers: What role do migrants play in your region? | Population |
| Organisation for Economic Co-operation and Development (OECD) | Health at a Glance: Europe 2020: State of in the EU Cycle | Intervention |
| Organisation for Economic Co-operation and Development (OECD) | Georgia: Health Systems in Action | Intervention |
| Organisation for Economic Co-operation and Development (OECD) | Kyrgyzstan: Health Systems in Action | Intervention |
| Organisation for Economic Co-operation and Development (OECD) | Uzbekistan: Health Systems in Action | Intervention |
| Organisation for Economic Co-operation and Development (OECD) | Beyond Containment: Health systems responses to Covid-19 in the OECD | Intervention |
| Organisation for Economic Co-operation and Development (OECD) | Finland: Country Health Profile 2021 | Intervention |
| Organisation for Economic Co-operation and Development (OECD) | State of health: Compendium Report | Intervention |
| Organisation for Economic Co-operation and Development (OECD) | Women at the core of the fight against COVID-19 crisis | Population |
| Organisation for Economic Co-operation and Development (OECD) | Workforce and Safety in Long-Term Care during the COVID-19 pandemic | Intervention |
| Organisation for Economic Co-operation and Development (OECD) | Netherlands – Country Health Profile 2021 | Intervention |
| Organisation for Economic Co-operation and Development (OECD) | Rising from the COVID-19 crisis: Policy responses in the long-term care sector | Population |
| Organisation for Economic Co-operation and Development (OECD) | Ukraine: Health Systems in Action | Intervention |
| Organisation for Economic Co-operation and Development (OECD) | Germany: Health system review. Health Systems in Transition, 2020 | Intervention |
| Organisation for Economic Co-operation and Development (OECD) | Slovenia: Health System Review | Intervention |
| Organisation for Economic Co-operation and Development (OECD) | Norway: health system review, Health Systems in Transition | Context |
| PAHO | Pan American Health Organization Response to COVID-19 in the Americas | Population |
| Rechel, B | How to enhance the integration of primary care and public health | Context |
| Richardson et.al | Keeping what works: remote consultations during the COVID-19 pandemic | Population |
| Thomas et.al | Strengthening health systems resilience: key concepts and strategies | Type of document |
